# Immunological pathogenesis of Bovine *E. coli* infection in a model of *C. elegans*

**DOI:** 10.1186/s12866-022-02733-5

**Published:** 2022-12-20

**Authors:** Hao Peng, Huili Bai, Yan Pan, Jun Li, Zhe Pei, Yuying Liao, Cuilan Wu, Changting Li, Li Tao, Shuhong Zhong, Chunxia Ma, Zhongwei Chen, Xiaoning Li, Yu Gong, Leping Wang, Fengsheng Li

**Affiliations:** 1 Guangxi Key Laboratory of Veterinary Biotechnology, Guangxi Veterinary Research Institute, Nanning, 530001 China; 2 Guangxi Agricultural Vocational University, Nanning, China; 3 The City College of New York, New York, USA; 4 Animal Science and Technology Station of Guizhou, Guiyang, China

**Keywords:** *Caenorhabditis elegans (C. elegans)*, Bovine, *Escherichia coli (E. coli)*, Pathogenicity, Innate immunity

## Abstract

**Background:**

Cattle industry is critical for China’s livestock industry, whereas *E. coli* infection and relevant diseases could lead huge economic loss. Traditional mammalian models would be costly, time consuming and complicated to study pathological changes of bovine *E. coli*. There is an urgent need for a simple but efficient animal model to quantitatively evaluate the pathological changes of bovine-derived *E. coli* in vivo. *Caenorhabditis elegans* (*C. elegans*) has a broad host range of diverse *E. coli* strains with advantages, including a short life cycle, a simple structure, a transparent body which is easily visualized, a well-studied genetic map, an intrinsic immune system which is conservable with more complicated mammalians.

**Results:**

Here, we considered that O126 was the dominant serotype, and a total of 19 virulence factors were identified from 41 common *E. coli* virulence factors. Different *E. coli* strains with diverse pathogenicity strengths were tested in *C. elegans* in *E. coli* with higher pathogenicity (EC3/10), *Nsy-1*, *Sek-1* and *Pmk-1* of the p38 MAPK signaling pathway cascade and the expression of the antimicrobial peptides *Abf-3* and *Clec-60* were significantly up-regulated comparing with other groups. *E. coli* with lower pathogenicity (EC5/13) only activated the expression of *Nsy-1* and *Sek-1* genes in the p38 MAPK signaling pathway, Additionally, both groups of *E. coli* strains caused significant upregulation of the antimicrobial peptide *Spp-1*.

**Conclusion:**

Thirteen *E. coli* strains showed diverse pathogenicity in nematodes and the detection rate of virulence factors did not corresponding to the virulence in nematodes, indicating complex pathogenicity mechanisms. We approved that *C. elegans* is a fast and convenient detection model for pathogenic bacteria virulence examinations.

**Supplementary Information:**

The online version contains supplementary material available at 10.1186/s12866-022-02733-5.

## Background

Bovine colibacillosis is a bacterial disease caused by the pathogenic *Escherichia coli (E. coli)*, which is commonly found in bovine [1, 2]. Because of the disease rapid transmission and widespread, it often leads to mass animal mortality in cattle farms and brings significant economic damage to the cattle industry. More seriously, *E. coli* disease as a zoonotic disease is easily transmitted from cattle to human through raw, undercooked beef, unsterilized dairy products, etc., posing a great threat to human health, which becoming a public health risk [3–6]. *E. coli* often co-acts with other bacteria, viruses, parasites, etc. to cause mixed infections [7–9]. Although the virulence role of *E. coli* isolated and obtained in this case is unknown in animals, and it is possible that it is not the main reason causing animal diseases or even death, but without knowing its detailed pathogenicity, it could cause misinformation that hidden the true sources of the animal infectious diseases. Currently, all in vitro methods for determining *E. coli* virulence requires either the identification of O serotypes or the detection of virulence factors. However, *E. coli* has a high genetic diversity with multiple genotypic and phenotypic changes [10], whereas identification of serotypes can only serve as a reference for *E. coli* virulence but cannot be used as a prediction evidence for the clinical pathogenic toxicity of *E. coli*. Additionally, *E. coli* has numerous virulence factors: dozens of them may associate with more than one diseases [11–14]. Meanwhile *E. coli* strains carrying the same virulence factors may have diverse levels of pathogenicity [15], representing complex pathogenesis of *E. coli*. Other probabilities include some virulence factors in particular *E. coli* strains that either without pathogenicity, or couldn’t been identified by current detecting tools [16], which ultimately leads to a significant difference between in vitro data and clinical pathogenicity results. Even worse, misguided by the incorrect in vitro data, people may get the wrong conclusion that *E. coli* strains with more virulence factors maybe even less toxicity than those with fewer virulence factors. Thus, in vitro data of *E. coli* serotypes and virulence factors is not sufficient enough to predict the real toxicity of *E. coli*, therefore it is a must to testing out in animal models to accurately verify its pathogenicity [17–19].

*Caenorhabditis elegans (C. elegans)* has a transparent body that is visible to the naked eye, with all basic anatomical structures, with a life cycle of about 3 weeks, also with a completely sequenced genome database: all of which make it a simple and practical animal model that comparable but more effective than traditional mammalian models such as mice, rats [20–22]. The homology between *C. elegans* genome and other mammals are about 40%, which made it an important reference model for studying pathogens including bacteria, fungi and viruses induced infections [23–26]. With a highly conserved immune system, *C. elegans* is used to study the pathogenic immune responses of bacteria and mechanisms of immune defenses [27–29], particularly relevant immune signaling pathways and antimicrobial peptides. There are three major immune signaling pathways in *C. elegans*: TGF-β signaling pathway, p38 MAPK signaling pathway and insulin receptor-like signaling pathway, which has different roles in resistance to external adverse stimuli, respectively [30–32]. Antimicrobial peptides such as lysozyme genes, ASABF family, sphingolipid-activated protein family and lectins are also important components of intrinsic immune responses that play the same immunomodulatory role in nematodes and mammals [33, 34]. This study uses *C. elegans* to study effects of the bovine-derived *E. coli* on the intrinsic immunity of nematode organisms therefore constructing a bovine-derived *E. coli*-*C. elegans* infection model. Following that, the model was compared with the mouse model in evaluating virulence of multiple strains of bovine-derived *E. coli*. Additionally, the expression of key regulatory genes of the immune signaling pathways were also screened in *C. elegans* to identify critical genes with immune protection effects in responses to *E. coli* infection. The study tends to provide a theoretical foundation for the pathogenesis study which potentially will be applied in preventing bovine *E. coli* diseases.

The advantage of establishment of a model of bovine *E. coli* in *Caenorhabditis elegans* is that the pathogenic effect of *E. coli* in *Caenorhabditis elegans* could intuitively reflect its true pathogenicity in living animals, which greatly solved the problem of inaccuracy in judging the pathogenicity of *Escherichia coli* by in vitro detection of *E. coli* serotypes, virulence factors and other indicators. It not only saved energy, time and test costs, but also could screen out pathogenic *E.coli* easily and quickly, which provided a great idea for the prevention and treatment of bovine colibacillosis, creating a precedent for the use of *Caenorhabditis elegans* to study bovine colibacillosis in China. This method is used as a preliminary screening tool for pathogenic bacteria, compared with large mammal cattle, it still cannot completely replace its role, which needs to be explained.

## Results

### Mouse experiments demonstrate differential pathogenicity of *E. coli* serotypes

The results of the pathogenicity test in mice are summarized in Table 1 (see Additional file 1: figure file 1). Eleven out of the thirteen *E. coli* strains were highly pathogenic to mice, with a lethality of 40-100%. The pathological features of mice were mainly pulmonary hemorrhAge and diarrhea, while two strains of *E. coli* were not lethal when testing in mice, with mainly manifesting as depression and loss of appetite only.

**Table 1:** Results of mouse pathogenicity test

Serotype	Pathological Changes	Numbers of Deaths	Lethality Rate/%
O127	Pulmonary hemorrhage, Diarrhea	3/5	60
O126	Pulmonary hemorrhage, Diarrhea	3/5	60
O44	Pulmonary hemorrhage，Hepatomegaly；Jaundice	5/5	100
O55	Pulmonary hemorrhage，Diarrhea	3/5	60
O20	Depression，Appetite loss，Gathering	0/5	0
O86	Diarrhea，Hemorrhagic gastroenteritis	4/5	80
O126	Diarrhea，Sepsis	4/5	80
O126	Diarrhea，Sepsis	4/5	80
O126	Diarrhea，Sepsis	4/5	80
O44	Diarrhea，hemorrhagic gastroenteritis	5/5	100
O15	Pulmonary hemorrhage，Diarrhea	3/5	60
O20	Pulmonary hemorrhage，(Hepatic hemorrhage)，Diarrhea	3/5	60
O7	Depression，Appetite loss	0/5	0

### Measuring the outcomes of virulence factors for *E. coli*

The detection rates of virulence factors of *E. coli* and carrying multiple virulence factors are shown in Tables 2 and 3 (see Additional file 1: figure file 1). After reviewing all detected *E. coli* virulence factors and pathogenicity outcomes of nematodes, these results showed that *E. coli* strains carrying a high number of virulence factors did not represent the strongest pathogenicity to neither nematodes nor mice. Therefore, we speculated that the numerous virulence factors of *E. coli* should have complex expression mechanisms. This lacking of correlation may indicate some undetected virulence factors. Another possibility could be that particular *E. coli* strain carrying some virulence factors but without expression.

**Table 2:** Detection rates of *E. coli* virulence factors

virulence factors	Number of detected strains	Detection rate/%
irp2	5	38.46
FyuA	5	38.46
Stx1	10	76.92
h1yA	9	69.23
SepA	2	15.38
987P	2	15.38
eaeA	4	30.77
escV	8	61.54
ent	6	46.15
ipaH	1	7.69
astA	8	61.54
uidA	9	69.23
iucD	8	61.54
ompA	13	100
vat	1	7.69
phoA	13	100
K88	1	7.69
CS31A	7	53.85
EAST1	3	23.08

**Table 3:** multiple virulence factors carried by *E. coli*

Number of virulence factors	Number of detected strains	Detection rate/%
13	2	15.38
12	1	7.69
11	1	7.69
10	1	7.69
9	4	30.77
8	2	15.38
6	1	7.69

### Cloning results of *E. coli* virulence factors

Each solution sample with positive testing result was sequenced following by BLAST comparison by using GenBank database (the registration number and homologous results are summarized in Table 4) (see Additional file 1: figure file 1). All homology results of corresponding virulence factors are over 96%, indicating that the PCR amplified products has the correct corresponding virulence factor of each sample.

**Table 4:** Results of virulence factor homologous

virulence factors	percent of homology /%	GeneBank Registration Number
irp2	100	CP028305
FyuA	99.0	KT825927
K88	99.5	CP015228
987P	98.71	JX987525
Stx1	99.38	MG986485
eaeA	97.56	JQ996412
h1yA	99.0	MF781079
SepA	96.2	Z49933.1
EscV	97.6	HQ428080
ent	98.1	AJ277443
ipaH	99.4	MG825757
astA	99.57	MN125562
uidA	100	CP043199
iucD	99.13	JX466843
ompA	100	FJ217631
phoA	100	MH469676
vat	99.7	KR094957
CS31A	99.5	M96174
EAST1	99.35	AB042003

### Results of nematode survival tests after *E. coli* infections

**Fig. 1:**
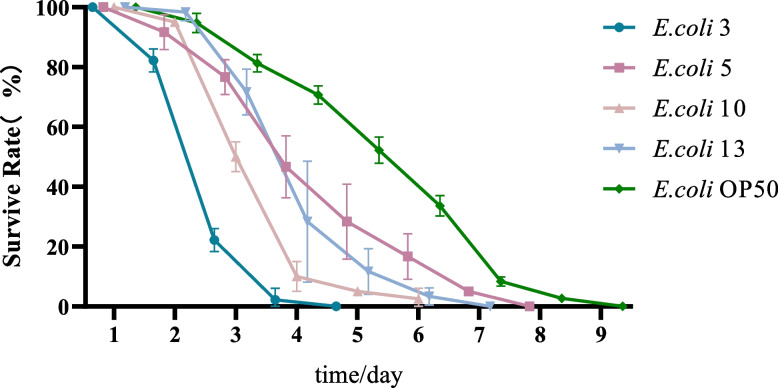
The survival rates of *C. elegans* on consecutive time-points after a particular *E.coli* strain infection. Figure legend: L4 stage wild-type N2 *C. elegans* were used as model animals. Three duplicates (20 nematodes each, total *n* = 60) were carried out in this study. Testing animals were transferred to fresh plates daily and stimulated with a picking needle for their responses. The number of survival nematodes was recorded. The survival rate of nematodes = number of live worms per day/60 × 100%. In Fig. 1, the dark blue line represents those nematodes fed on *E. coli* 3. The pink line represents nematodes fed *E. coli* 10. The purple line represents nematodes feeding on *E. coli* 5. The light blue line represents nematodes feeding on *E. coli* 13. The green line represents nematodes feeding on *E. coli* OP50

The survival rates of *C. elegans* on consecutive time-points after a particular *E.coli* strain infection is shown in Fig. 1. To determine the pathogenicity strength of each *E.coli* strain on nematodes, all thirteen *E.coli* strains which isolated from cattle were tested, and the data were clustered into three categories: The strong, the medium and the weak groups. The pathogenicity level was evaluated by survival testing results of nematodes, which will be detailed described below. All data were summarized in Table 5 (see Additional file 1: figure file 1).

**Table 5:** The evaluation of *E. coli* pathogenicity to nematode

Time of nematode infection	Weak pathogenicity /%	Medium pathogenicity /%	High pathogenicity /%
Day1	0	0 ~ 10	0 ~ 5
Day2	0 ~ 5	5 ~ 20	20 ~ 25
Day3	5 ~ 10	10 ~ 48	50 ~ 53
Day4	20 ~ 30	35 ~ 70	80 ~ 85
Day5	45 ~ 50	65 ~ 87	80 ~ 90
Day6	50 ~ 60	75 ~ 95	93 ~ 95
Day7	55 ~ 80	85 ~ 100	95 ~ 100
Day8	65 ~ 85	0	0
Day9	75 ~ 90	0	0
Day10	80 ~ 95	0	0
Day11	90 ~ 100	0	0

When *E. coli* infected *C. elegans*, the longest survival time and half-lethal time of nematodes could be observed, together with their mortality rates and total survival times. Among all tested *E. coli* strains, EC 3 and EC10 were the most pathogenic to nematodes: the longest survival time of nematodes which infected with these two strains were seven days and nine days respectively with an averAge of three days’ half-lethal time. Comparing to other testing samples, the survival rates of nematodes infected with EC3 and EC10 were droped to 50% on the third day. Therefore, these strains were considered to have a highly pathogenic level. To the contrary, EC 5 and EC13 were the least pathogenic to nematodes, with a maximum survival time of eleven days and ten days respectively together with a half mortality time of six days and five days. These strains were considered to have the least pathogenic to nematodes. For the other nine *E. coli* strains, maximum survival time of infected nematodes were between seven to ten days with a half mortality time between 3.1 to 4.5 days. These *E. coli* strains were considered as moderately pathogenic ones. Generally, the weaker the pathogenicity of *E. coli*, the longer the survival time and the half time to death of nematodes. But for *E. coli* strains with high and moderate pathogenicity, the maximum survival time of nematodes only show moderate variability. Additionally, O157, the most toxicity strain was used as the positive control in this experiment. Survival rates of nematodes which infected with O157-positive controls declined fastest, which also shown the highest lethality rates. Therefore, all tested *E. coli* strains were less lethal comparing with O157, with a downward trend in nematode survival rate compared to the positive controls (O157 infected). As data shown in Table 5, the pathogenicity of *E. coli* remarkably diverse after the second day of testing. Nematode lethality was: 1) ≤5% for the weakly pathogenic strains; 2) between 5 and 20% for the moderately pathogenic strains; and 3) ≥20% for the highly pathogenic strains. At the third day, nematode lethality was between 5 to 10% for the weakly pathogenic strains, which was between 10 to 48% for the moderately pathogenic strains, while this range was greater than 50% for the highly pathogenic strains. At day 4, the lethality of the weakly pathogenic strains was between 20 and 30%; the lethality of the moderately pathogenic strains was between 30 - 70%; and the lethality of the highly pathogenic strains was ≥85%. These results represented that pathogenicity variations of *E. coli* was mainly affected between 2 and 4 days after the infection of nematodes.

### Comparison results of *E. coli* pathogenicity by using mice and nematodes

We found those highly pathogenic *E. coli* strains in mice also have the highest lethality in nematodes (100%). Similarly, *E. coli* strains that were weakly pathogenic to mice are also less lethality to nematodes (0%). It indicated that mice and nematodes have very similar pathogenicity responses of *E. coli* infections. Therefore, nematodes may able to be considered as an alternative model of rodents to study *E. coli* infections.

### *E. coli* infection affects nematode immune signaling pathways

**Fig. 2:**
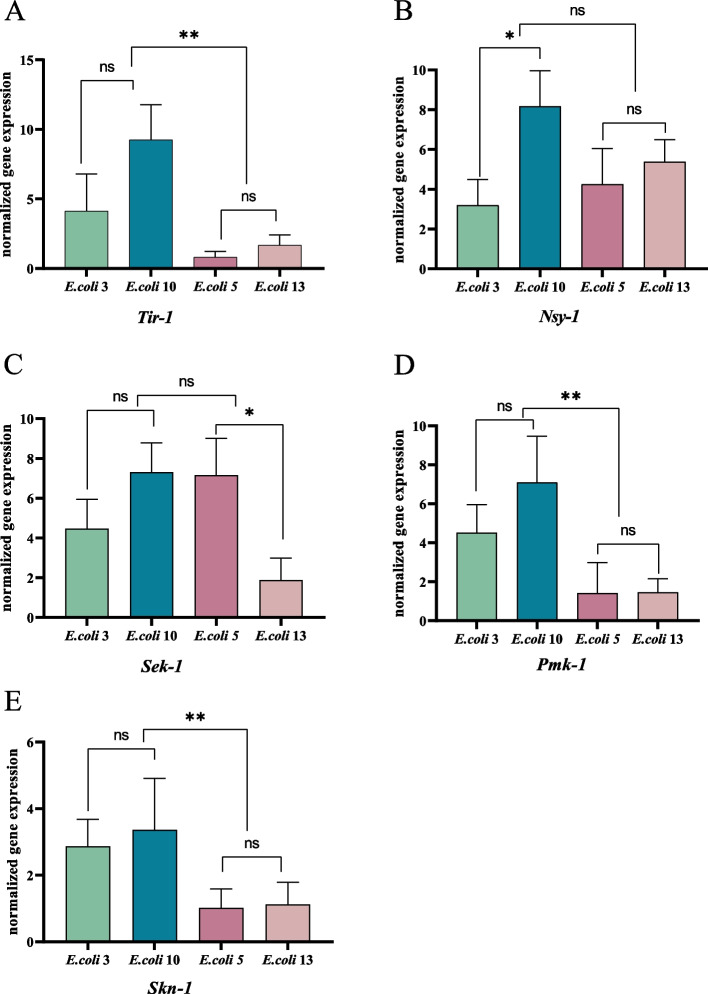
Expression of immune genes in p38MAPK signaling pathway. Figure legend: the graph-**a** represents *tir-1* gene expression changes in nematode after infected with *E. coli* strains of diverse pathogenicity. The graph-**b** represents changes of *nsy-1* gene expression. The graph-**c** represents changes of *sek-1* gene expression. The graph-**d** represents changes in *pmk-1* gene expression. The graph-**e** represents changes of *skn-1* gene expression. Differences within and between groups were calculated separately and plotting by GraphPad Prism software. ‘ns’: non-significant differences (*P* ≥ 0.05). *: significant difference (*P* ≤ 0.05). **: significant difference (*P* ≤ 0.01). ***: significant difference (*P* ≤ 0.001). ****: significant difference (*P* ≤ 0.0001)

The expression of immune genes in p38 MAPK signaling pathwayp38 MAPK is a critical signaling pathway in nematodes’ immune system. This study observed significant expression level changes among multiple critical genes in p38 MAPK signaling pathway, which could be the result of immune reactions to resist the infection of pathogenic bacteria and as a response to external stimuli. As shown in Fig. 2 (see Additional file 1: figure file 2), *Tir-1*, *Nsy-1*, *Sek-1* and *Pmk-1* genes, which belong to the *Tir-1* → *Nsy-1* → *Sek-1* → *Pmk-1* cascades of the p38MAPK signaling pathway, were all upregulated after the infection of either EC3 or EC10. When tested nematodes which infected with EC3, *Sek-1* and *Pmk-1* genes were both significantly up-regulated for 4.47-folds and 4.53-folds respectively, together with 4.14-folds of *Tir-1* and 3.2-folds of *Nsy-1*. For nematodes which infected with EC10, *Tir-1*(9.25-folds) and *Nsy-1*(8.17-folds) were the highest up-regulated genes, together with *Sek-1*(6.82-folds) and *Pmk-1*(7.11-folds). Compared with the EC3 group, the *Tir-1*, *Nsy-1*, *Sek-1*, and *Pmk-1* genes were significantly upregulated in the EC10 infected group, while no significant change of *Skn-1* was observed. Interestingly, the same gene *Skn-1* was 2.87-folds upregulated in the EC3 infected group, suggesting diverse immune pathways could be affected by different *E. coli* strains. For nematodes infected with weak pathogenicity *E. coli* strains: only *Nsy-1*(4.26-folds) and *Sek-1*(7.16-folds) were up-regulated after EC5 infection; while only *Nsy-1* was 5.39-folds up-regulated among nematodes infected with EC13. No significant changes in other immune genes in these groups.

#### Expression of immune genes of TGF-β signaling pathway

**Fig. 3:**
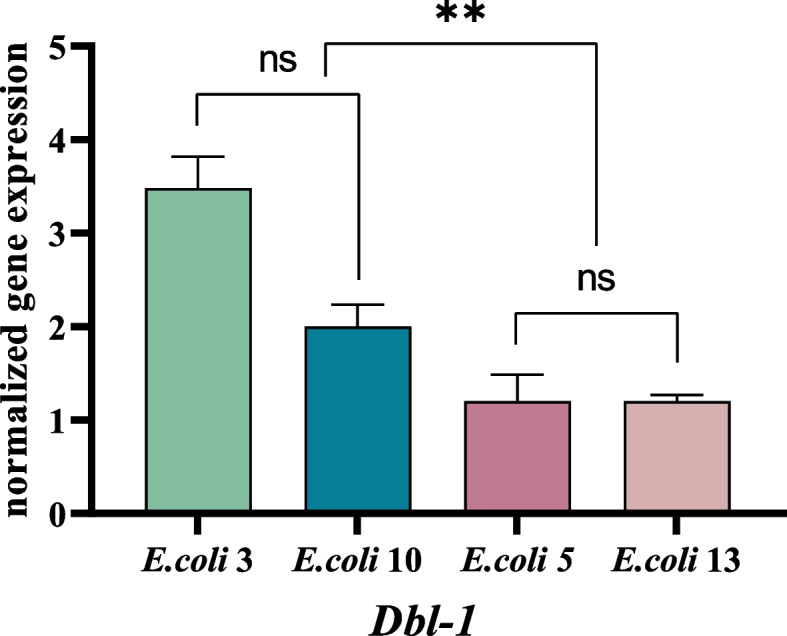
Expression of *Dbl-1* of TGF-β signaling pathway. Figure Legend:‘ns’: non-significant differences (*P* ≥ 0.05). *: significant difference (*P* ≤ 0.05). **: significant difference (*P* ≤ 0.01). ***: significant difference (*P* ≤ 0.001). ****: significant difference (*P* ≤ 0.0001)

Among nematodes infected by *E. coli* EC3 or EC10, the expression of *Dbl-1* gene of the TGF-β signaling pathway was upregulated 3.48 folds (EC3) and 2.0 folds (EC10). For weakly pathogenic *E. coli* strains, no significant change of *Dbl-1* was detected after infection (Fig. 3) (see Additional file 1: figure file 2).

#### Expression of immune genes of insulin-like signaling pathway

**Fig. 4:**
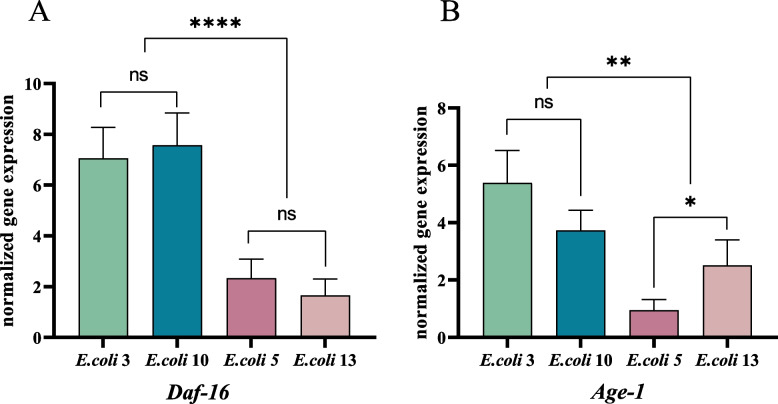
Expression of immune genes of insulin-like signaling pathway. Figure legend: **a** Two strains, the *E. coli 3* and 10, both highly express *Daf-16* gene after infection while this gene expresses relevant low in the other two strains, *E. coli* 5 and 13. There is no significant variances between *E. coli 3* and 10, or between *E. coli 5* and 13. But the *Daf-16* gene expression was significantly different among the two groups. **b** The change in *Age-1* gene expression is not significant after the infection of *E. coli* 3 and 10, but are statistical significantly differently either between *E. coli* 5 and 13, or among *E. coli* 3 and *E. coli* 5, 13 and also among *E. coli* 10 and *E. coli* 5, 13. It reflecting different pathogenicity among *E. coli* strains. ‘ns’: non-significant differences (*P* ≥ 0.05). *: significant difference (*P* ≤ 0.05). **: significant difference (*P* ≤ 0.01). ***: significant difference (P ≤ 0.01)

*Daf-16* and *Age-1* are both critical genes of the insulin-like signaling pathway, which controls the lifespan of nematodes. We observed that *Daf-16*(E3:1.6-folds, E10:7.57-fold) and *Age-1*(E3:5.39-folds, E10: 3.73-folds) were both up-regulated in nematodes after the infection of *E. coli* EC3 and EC10 (Fig. 4) (see Additional file 1: figure file 2). For infection of weak pathogenic *E. coli* strains (EC5, EC13), Daf-16 was up-regulated only in EC5 infected group (EC5: 2.33-fold), while *Age-1* shown an insignificant trend of slightly down-regulation in the E5 infected group but was up-regulated 2.51-fold among the EC13 infected ones.

#### Expression of antimicrobial peptide genes

**Fig. 5:**
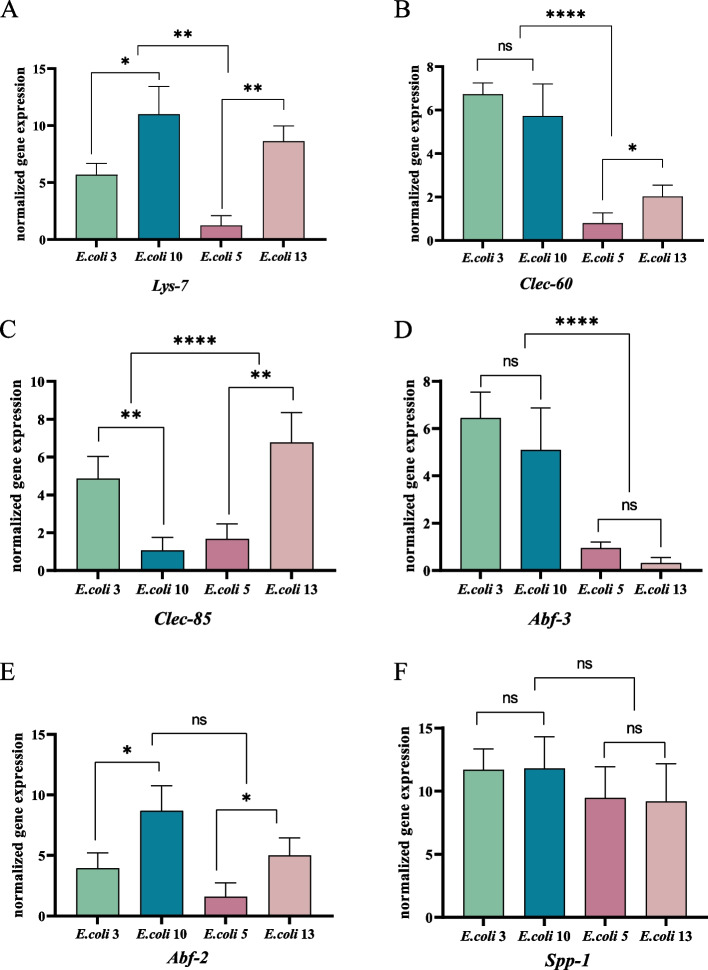
Expression of antimicrobial peptide genes. Figure legend: **a** represents the expression change of antimicrobial peptide gene *Lys-7* in nematode after infected with *E. coli* strains of different pathogenicity. **b** the expression changes of *Clec-60* gene after *E. coli* infection. **c** the expression changes of *Clec-85* infection. **d** the expression changes of *Abf-3* after infection. **E** the expression changes of *Abf-2* gene after *E. coli* infection in nematodes. **F** the expression changes of *Spp-1* gene after infection. ‘ns’: non-significant differences (*P* ≥ 0.05). *: significant difference (*P* ≤ 0.05). **: significant difference (*P* ≤ 0.01). ***: significant difference (*P* ≤ 0.001). ****: significant difference (*P* ≤ 0.0001)

In Fig. 5 (see Additional file 1: figure file 2), our study demonstrated that the nematode antimicrobial peptide genes *Spp-1 (Caenopore-1)*、*Abf-2*、*Clec-85*、*Lys-7* were all upregulated after *E. coli* infection. Particularly, for those infected by the strongest pathogenicity strains, Spp-1 was significantly upregulated (EC3: 11.7 folds, EC10: 11.8 folds). Among those infected by lesser pathogenic strains such as EC5 and EC13, the four antimicrobial peptide genes were also moderately upregulated for around 4.99- 9.45 folds. Interestingly, some other antimicrobial peptide genes such as *Abf-3*、*Clec-60* shown different responses between groups: they were strongly upregulated after EC3 and EC10 infection but down regulated by those weaker pathogenic strains with an expressions ranging of 0.17- 0.98 fold of uninfected nematodes.

## Discussion

The results of this study showed that the virulence determined by the in vitro PCR technique for *E. coli* virulence factors did not correspond to the virulence of *E. coli* expressed in *C. elegans*. It could due to the complex pathogenesis of *E. coli*, whose ability to exert virulence on the host depends on the balance between the state of the host and the presence and expression of *E. coli* virulence factors. Therefore, the in vitro results of virulence factors cannot reflect the in vivo conditions and the complex immune reactions after *E. coli* infection [35–37]. In addition, although O serotyping is important to determine the virulence of *E. coli* [38, 39], the method has its own limitations: antisera produced against specific O groups may cross-react, and another being that some *E. coli* may lack O antigens and cannot be isolated or identified [40]. The above discussion shows that in vitro detection of *E. coli* virulence factors and identification of O serotypes cannot reflect the real pathogenicity of *E. coli* in cattles, the in vitro detection data to determine the virulence of clinically isolated *E. coli* also lacks accuracy, which must be confirmed by in vivo tests using animal models to verify their virulence. Since it could be expensive and not be practical to use cattle as preliminary testing animals, animal models could offer the possibility of establishing causality links, allowing robust interpretations of the real influence of one system on the other [41, 42]. So we choosed *C. elegans* and mice to study the virulence of bovine pathogenic bacteria, both of which were very typical model organisms. Our results demonstrated that all 13 strains of bovine-derived *E. coli* in this study showed the same lethality in *C. elegans* and *Kunming* mice, indicating that the virulence of *E. coli* was the same in nematodes and mice, and the bovine-derived *E. coli* infection model was successfully established in *C.elegans*. Moreover, a criterion for determining the strong, medium and weak pathogenicity of *E. coli* to nematodes was also established by comparing the half lethality time of 13 *E. coli* strains and the nematodes’ survival rates, which provided a reference standard for the subsequent classification of the pathogenicity of clinical isolated *E. coli* strains. Since both nematodes and mice could get the same test results, it reflected the importance of predicting the success of a trial at an early stage of research, as it involves time and spending. An SPF mouse needs ¥12 or more and subsequent experimental animal handling fee is also required. The mouse test could get result in one week but cannot guarantee that the pathogenic *E. coli* can be accurately screened. *C. elegans* perfectly fits such requirements:it is a valuable tool in pathogenicity and can predict pathogenicity outcomes in mammals. It is inexhaustible and can get result in 2 ~ 3 days. The most important is that mice are mammals, and there are ethical issues involved in the use of them, while *C. elegans* completely ignores this issue and guarantees that results can be obtained rapidly [43, 44]. So we concluded that *C. elegans* is more suitable than mice to study the virulence of pathogenic bacteria.

The immune genes and antimicrobial peptides of *C. elegans* were detected by fluorescence quantitative PCR. The effects of different pathogenic *E. coli* strains were also analyzed by observing multiple critical genes from nematode immune signaling pathways, which provided a strong theoretical foundations for clinical prevention and the potential anti-infection treatment. The fluorescence quantitative PCR results of this study showed that *E. coli* with different pathogenicity differed significantly in the regulation of nematode immune gene and antimicrobial peptide expression. In the TGF-β signaling pathway, *E. coli* with high pathogenicity significantly upregulated nematode *Dbl-1* gene expression, while *E. coli* with weak pathogenicity had almost no effect on *Dbl-1* expression, indicating that *Dbl-1* expression was directly influenced by the pathogenicity of *E. coli* [45]. *Dbl-1* is an important ligand of the TGF-β signaling pathway in *Caenorhabditis elegans* and is expressed in the pharynx, subcutaneous tissues and intestine *Dbl-1* is a homolog of nematode bone morphogenetic protein 2/4 (BMP2/4), which is involved in regulating nematode growth and development and intrinsic immunity, and is essential for sensory-motor responses [46, 47]. Since *Caenorhabditis elegans* feeds on bacteria, it is able to learn and recognize the odor of different bacteria, and is able to regulate its interaction with pathogenic bacteria through olfactory learning for those bacteria that are pathogenic or survival threatening [48, 49], and *Dbl-1* mutants are usually more sensitive to pathogens, thus it can be inferred that this gene plays an extremely important role in defending against harmful bacteria [50] . Zhang’s findings suggest that nematodes do not induce olfactory aversion when exposed to non-pathogenic bacteria such as *Pseudomonas fluorescens*, while short-term exposure to the pathogenic bacterium *Serratia marcescens* induces aversion learning in adults [51]. Moreover, *Dbl-1*-deficient mutants are defective in avoiding learning of the pathogenic bacterium *Serratia marcescens*, but the introduction of DNA from the *Dbl-1* genome again rescues the mutant’s defect in learning [51] . In addition, *Dbl-1* is important for maintaining the natural abundance of Enterobacteriaceae members in the natural microbiota of *Caenorhabditis elegans*, and deletion of the *Dbl-1* gene changes the role of these bacteria from symbiotic to pathogenic [52]. The above studies suggest that this gene is essential for the learned behavior of nematodes in the face of potentially harmful bacterial food for aversive olfaction. This is consistent with the results of the present experiment: when a highly pathogenic *E. coli* infected the nematode, the organism recognized the bacterium as a harmful bacterium and triggered a significant upregulation of the *Dbl-1* gene to enhance the ability of the organism to escape from the harmful bacterium, whereas a weakly pathogenic *E. coli* was minimally lethal to the nematode, invading the organism, the pathogen was not recognized as a harmful bacterium, and did not trigger olfactory aversion to the pathogen. Thus, no upregulation of *Dbl-1* was activated to escape the pathogen.

The p38 mitogen-activated protein kinase (p38 MAPK) signaling pathway of *Caenorhabditis elegans* is highly conserved with mammals, involved in responses to various physiological stimuli and environmental stresses, and plays an important role in the intrinsic immunity of nematodes. The results of this experiment showed that in the *TIR-1* → *NSY-1* → *SEK-1* → *PMK-1* → *SKN-1* cascade of the p38 MAPK signaling pathway, the expression of each immune gene in the nematode p38 MAPK signaling pathway was upregulated to different degrees by pathogenic *E. coli*, among which the upstream *Tir-1*, Nsy-1, Sek-1 and *Pmk-1* were upregulated The up-regulation of *Tir-1*, *Nsy-1*, *Sek-1* and *Pmk-1* was obvious, and the up-regulation of terminal *Skn-1* was relatively small, but it was still higher than the expression of *Skn-1* in *E. coli* group with weak pathogenicity. The weakly pathogenic *E. coli* only activated the upregulation of midstream *Nsy-1* and *Sek-1*, and did not activate the expression of *Pmk-1* and *Skn-1*, indicating that *Nsy-1* and *Sek-1* are more sensitive in response to *E. coli* infection, which is consistent with the result of Dennis’ screening of *Nsy-1* and *Sek-1*, two pathogen resistance genes, from a variety of nematode mutants with increased susceptibility to *P. aeruginosa* lethality, suggesting that both are essential genes for resistance to pathogenic bacteria and play an important role in resistance to pathogen infection [53]. *Nsy-1* and *Sek-1* are homologs of the mammalian apoptosis signal-regulated kinases *ASK1* and *MKK3/6*, respectively. *Nsy-1* is a direct activator of *Sek-1* and is expressed in many tissue types, including the intestine [53]. p38 MAPK *PMK-1* is activated by *TIR-1*, *MAPKKK NSY-1* and *MAPKK SEK-1* and is a key gene in resistance to pathogenic bacterial infection. Bolz et al. were able to enhance *Yersinia pestis* susceptibility by mutation or RNAi inhibition of *Pmk-1* / p38, indicating an important role of *Pmk-1* / p38-regulated immune-related effectors in resistance to *Yersinia pestis* [54]. In mediating responses to pathogens, Salmonella-induced programmed cell death in *Caenorhabditis elegans* hosts appears to be associated with protective responses, and inactivation of RNAi *Pmk-1* blocks Salmonella-induced programmed cell death. The transcription factor *Skn-1* is a homolog of the mammalian Nrf protein, and the DNA binding mechanism causes *Skn-1* to be less active than other proteins, exhibiting nuclear translocation only when activated by *Pmk-1* to function as an activator of the oxidative stress response. The inactivity of *Skn-1* clarifies the relatively small upregulation of *Skn-1* in this study and the fact that *E. coli* with high pathogenicity are more sensitive to oxidative stress than *E. coli* with low pathogenicity. The inactivity of *Skn-1* clarified that the up-regulation of *Skn-1* in this study was more pronounced in *E. coli* with relatively low pathogenicity than in *E. coli* with low pathogenicity.

The insulin-like signaling is an evolutionarily conserved pathway with significant functions in phosphorylation. Insulin-like signaling is well known in controlling metabolism and lifespan growth, which also regulates the immune responses of *C. elegans*. The *Daf-16* is a homolog of the FOXO family in *C. elegans*, known as the primary transcription factor of nematodes’ insulin-like cascades and the main downstream target of the insulin-like receptor *DAF-2* [55]. The *Daf-16* is also essential for maintaining nematode longevity in both wild-type and germline-deficient contexts. In *C. elegans*, either overexpressing *Daf-16* or simply increasing *Daf-16* protein activities could increase their resistance to various pathogens.

*Age-1* is a homologue of the phosphatidylinositol-3-hydroxyl kinase PI3K, which plays a role to some extent in the formation of the Dauer phase, resistance and lifespan direction in nematodes. *Age-1* may reduce fertility in hermaphrodites or other unknown metabolic/ physiological changes that has a lifespan extension effect. Mutations of *Age-1* causes nematode growth to stagnate at the Dauer stage, shifting metabolism to fat accumulation and extending its lifespan, which similar to the function of mammalian insulin in metabolic regulation. In this experiment, EC3 with high pathogenicity stimulated the expression of nematode *Age-1*, while EC10 increased the expression of *Daf-16* and *Age-1*. Whereas weak pathogenicity strains EC5 and EC13 induced lower expressions of nematode *Daf-16* and *Age-1* genes when comparing with high pathogenicity *E. coli* infected groups. It indicates that the pathogenicity of *E. coli* directly affected the expression of life span genes in nematodes.

Antimicrobial peptides involve in antimicrobial activity and have important roles in innate immunity of *C. elegans* [56]. As a respond to pathogens, *C. elegans* produces specific proteins such as C-type lectins, hydrolases, Lysozyme and *Spp-1*. The caenopore-1 protein is encoded by *Spp-1* gene and specifically expressed in the intestine of *C. elegans*. The Salmonella infection could strongly enhance the expression *Spp-1* and inhibit Salmonella reproduction [57]. When infected with Enterotoxigenic *Escherichia coli* (ETEC), *C. elegans* with Spp-1 mutants shown a significantly shorter lifespan compared to wild types, indicating that *Spp-1* has an important role in defense ETEC. Antimicrobial factor (Abf) is an antimicrobial peptide identified in *Caenorhabditis elegans*, and six ABFs (*Abf-1* to 6) have been identified from *Caenorhabditis elegans* [58], which play a direct role in the intrinsic immunity of nematodes. Abf-3 is usually expressed in the intestine, and the results of this experiment indicate that the expression of *Spp-1* is more sensitive to the pathogenicity of *E. coli*, with different pathogenicity The expression of *Spp-1* was significantly up-regulated after the infection of nematodes by *E. coli* with different pathogenicity, which may play an early immune response at the early stage of the organism’s response to foreign pathogens, and is a rapid response and more sensitive defense mechanism. In contrast, the expression of *Abf-3* and *Clec-60* was positively correlated with the pathogenicity of *E. coli*, and the expression was significantly up-regulated under the aggression of *E. coli* with strong pathogenicity, but did not show significant changes for *E. coli* with weak pathogenicity, indicating that these two immune genes are susceptible to activation only when external adverse stimuli reach a certain level. It was shown that the expression level of the C-type lectin *Clec-60* was significantly reduced after *Pmk-1* or *Sek-1* deletion in response to Acinetobacter candida infection, indicating that the induction range of C-type lectin is highly correlated with the p38 MAPK pathway [31]. The expression of *Abf-2*, *Clec-85*, and *Lys-7* did not correlate significantly with the pathogenicity strength of *E. coli*. This study is the first time to isolate strains of bovine *E. coli* from East and West China, identifying their unique O-serotype types and evaluating their carriAge of virulence factors. Using these bovine *E. coli* strains, this study also established the first *C. elegans* pathogenic infection model to establish an in vivo system for efficient, convenient and rapid detection of virulence *E. coli* infections and to explore the immune responses of these strains among *C. elegans.* Limited by the time and resources, in this study only three general categories of *E. coli* were analyzed: the strong, moderate, and weak pathogenicity based on the survival rate and the half-lethal time of the nematodes. In future studies, we will generate a more detailed analyzing system which could including more features in testing the pathogenicity of *E. coli* strains and to analyzing their immune responses in *C. elegans*. In this study, only the most and least virulent *E. coli* strains were used, because we tend to explore the spectrum of immune reactivities for *C. elegans* and their regulatory effects by pushing the testing condition close to the boarder. For the next stage, we plan to extend our study to those bovine *E. coli* strains with moderate pathogenicity features, and plan to use both sets of data to create a relevant database of *C. elegans*. Additionally, there are numerous immune genes and pathways which functional in *E. coli* infections among nematodes, but with limited resources, only TGF-β, p38 MAPK and the insulin-like signaling pathways were studied together with some classical immune genes could be selected in this study based on previously reported results. We consider these pathways as hubs that controlling nematodes immune reactivities, particularly in immune defense functions. These genes and antimicrobial peptides can be better compared and discussed with previous studies.

Overall, this study demonstrated that *C. elegans* is an effective infection model in testing the immune responses of diverse kinds of bovine *E. coli* strains, although in-depth studies still needed to correlate their pathogenic toxicities and underlying immune processes. This set of results also illustrates the virulence of *E. coli* of bovine origin, and diverse molecular mechanisms that regulate the three immune signaling pathways. We expect this *C. elegans* model could not only be valuable in bovine sourced bacteria identification and immunology mechanism research, but also contribute for the future control of bovine diseases.

## Materials and methods

### Nematodes and strains

The test strain was wild-type *N*_2_ *Caenorhabditis elegans* and 70 4-week-old SPF-grade Kunming mice. Thirteen strains of *E. coli*isolated from bovine disease material collected from different regions of China in 2021-2022. The positive control for nematode was pathogenic E.coli O157. The negative control for nematode was OP50. *E.coli* strains were scribed on LB solid medium and incubated in a constant temperature incubator at 37 °C for 18 h. Individual colonies were picked into 5 mL LB broth and shaken in a shaker for 12 h.

### Pathogenicity test of *Escherichia coli* on mice

The cultured *E. coli* solution was diluted to 3.0 × 10^9^ CFU/mL after calculating the concentration of bacterial solution. Seventy Kunming mice were randomly divided into 14 groups of 5 mice each, and the mice in the test group were injected with 0.3 mL of 3.0 × 10^9^ CFU/mL bacterial dilution into abdominal cavity, and the mice in the control group were injected with 0.3 mL of sterile saline intraperitoneally. The above 14 groups of mice were kept separately and allowed to feed and drink freely. The time of death and the number of mice were observed every day. The mice that showed symptoms and died in the pathogenicity test were promptly dissected, and the lesion sites were collected aseptically and isolated for pathogenic bacteria. (All methods were carried out in accordance with relevant guidelines and regulations).

### Detection of *E. coli* virulence factors

DNA of 13 strains of *E. coli* was PCR amplified according to the synthesized primers, and the amplified PCR products were electrophoresed on a 1% agarose gel, followed by subsequent experiments. (The reaction system of PCR was listed in Additional file 2: Table S1).

### Cloning of *Escherichia coli* virulence factors

One percent agarose electrophoresis gel was prepared. After cooling, the amplified PCR product was subjected to agarose gel electrophoresis, electrophoresised for 20 minutes and then the single target DNA band was cut from the agarose gel and put into a clean centrifuge tube and weighed the gel to calculate the weight. One volume of Buffer PG was added to the centrifuge tube and incubate in a water bath at 50 °C, during which the centrifuge tube was gently turned upside down every 2-3 min until the sol was yellow to ensure that the gel was fully dissolved. If there were still undissolved gel pieces, added some more sol solution or keep it for a few minutes until the gel pieces were completely dissolved. 200 μl of Buffer PS was added to the Spin Columns DM in the collection tube, centrifuged at 13,000 rpm (~ 16,200×g) for 1 min, discard the waste liquid in the collection tube, and put the spin column back into the collection tube. The solution obtained in step 3 was added to the adsorption column that has been loaded into the collection tube, let stand at room temperature for 2 minutes, centrifuge at 13,000 rpm for 1 min, pour off the waste liquid in the collection tube, and put the adsorption column back into the collection tube. 450 μl Buffer PW was added to the adsorption column (which had been added absolute ethanol before), centrifuged at 13,000 rpm for 1 min, discard the waste liquid in the collection tube, and put the adsorption column back into the collection tube (repeated this step once). Centrifuged at 13,000 rpm for 1 min and discard the waste in the collection tube. Put the adsorption column into a new 1.5 ml centrifuge tube, 50 μl Buffer EB dropwise was added to the middle of the adsorption membrane, and left it at room temperature for 2 mins. The DNA solution was collected by centrifugation at 13,000 rpm for 1 minute.

The recovered product of the target fragment was connected to the pMD-18 T vector. The reaction system is shown as below: recovered product 1 μL, Solution I 5 μL, pMD-18 T Vector 1 μL, RNase free H_2_O 3 μL. After gentle mixing, ligation overnight at 16 °C.

The ligation product was transformed into DH5α competent cells according to the commercial instructions. Competent cells were taken and placed in ice bath, after the competent cells were thawed on ice, added the target DNA to the competent cell suspension, Gently mix and ice bath for 30 minutes. Heat shocked at 42 °C for 90 seconds, quickly transfer the centrifuge tube to an ice bath, and let stand on ice for 2-3 minutes. 900 μl of sterile LB medium was added to each centrifuge tube, mixed well, placed on a shaker at 37 °C, and incubated at 150 rpm for 45 minutes to recover the cells. 100 μl of transformed competent cells was taken and added into LB solid agar medium containing ampicillin, spreaded the cells evenly with a sterile coating rod until dry, inverted the plate, and cultivated overnight at 37 °C. A single colony was picked from the LB plate cultured overnight in ampicillin-resistant LB broth, placed in a constant temperature shaker at 37 °C for 2 h, and the bacterial solution was used as a template to carry out PCR amplification with 16S primer, electrophoresis, and the Bacteria that tested positive were sequenced.

### Pathogenicity test of *Escherichia coli*

The eggs were incubated in S-Basal medium at 20 °C, and transferred to NGM plates containing 60 mm of *E. coli* OP50 moss after hatching into L1 stage, and transferred to NGM plates with *E. coli* moss when growing into L4 stage nematodes, with 20 worms per plate and 3 replicates per strain, using *E. coli* OP50 as negative control and pathogenic *E. coli* O157 as positive control. The number of nematode survivors was observed and recorded daily.

### Effect of *Escherichia coli* on key genes of nematode immune signaling pathway

The two *E. coli* strains with the strongest pathogenicity to nematodes (EC3 and EC10, respectively) and the two strains with the weakest pathogenicity (EC5 and EC13, respectively) among the 13 *E. coli* strains were selected. The nematodes infected with *E. coli* in each group were collected every 24 h, washed with PBS and centrifuged, and the RNA of the nematodes was extracted according to the steps as below: Centrifuge at 12,000 rpm for 2 minutes, carefully aspirate the supernatant. 1 ml of absolute ethanol was added and vortex to mix. Centrifuge at 12,000 rpm for 2 minutes, discarded the supernatant carefully. Open the lid and incubated at room temperature or up to 37 °C for 10 minutes until no ethanol remains. 150 μl of Lysis Buffer 1 was added to resuspend the pellet; added 10 μl of proteinase K, and mixed by vortexing. Incubated at 56 °C for 15 minutes until the sample was completely dissolved. Incubated at 80 °C for 15 minutes. Briefly centrifuged to collect the solution on the tube wall to the bottom of the tube. 320 μl of Lysis Buffer 2 was added and vortex to mix thoroughly. All the solution obtained was added to the filter column that has been loaded into the collection tube. The filtrate was collected by centrifugation at 12,000 rpm for 1 minute. 720 μl of absolute ethanol was added and vortex to mix thoroughly. All the solution to the adsorption column that had been loaded into the collection tube. If the solution cannot be added at one time, it could be transferred in multiple times. Centrifuge at 12,000 rpm for 1 minute, discarded the waste liquid in the collection tube, and put the adsorption column back into the collection tube. 500 μl of washing buffer (with absolute ethanol added) was added to the adsorption column, centrifuged at 12,000 rpm for 1 minute, discarded the waste liquid in the collection tube, and put the adsorption column back into the collection tube. Centrifuged at 12,000 rpm for 2 minutes and discard the waste liquid in the collection tube. Allow the cartridge to dry at room temperature for several minutes. Placed the adsorption column in a new collection tube, 20-50 μl of elution buffer was added to the middle of the adsorption column, stood at room temperature for 2-5 minutes, centrifuged at 12,000 rpm for 1 minute, the RNA solution was collected and store at − 80 °C.

qPCR was performed after reverse transcription with the reaction procedure: 95 °C for 10 mins; 95 °C for 30 s, 60 °C for 1 min, 72 °C for 1 min, 40 cycles. Each sample were tested triplicate. The average relative quantitative (RQ) value for all target genes were calculated and get the geometric mean to get the mean stability as the norm. The normalized expression of each sample was calculated for each sample. All expression data were log2 transformed to get the standard deviation (SD) of each group and plotting together with the mean value in the bar-graph. The log2 transformed values were also used for further statistical analysis in the SPSS software (IBM).

### Statistical analysis

Statistical analysis were performed using SPSS 25.0 software. One-way analysis of variance (ANOVA) was performed for determination of differences in gene expression values between *E.coli* with the same virulence. When a significant difference was detected between the two strains of *E.coli*, Bonferroni post-hoc test was performed for determination of the differences. Unpaired t test was performed for determination of differences in gene expression between two groups of *E.coli* with different pathogenicity. The significance level for the obtained *p*-value was set at 0.05, 0.01, 0.001 and 0.0001.

## Supplementary Information

**Additional file 1.**

**Additional file 2.**

**Additional file 3.**

**Additional file 4.**

**Additional file 5.**

**Additional file 6.**

**Additional file 7.**

## Data Availability

There is no data submitted to the repository for this study,but all data and material are available upon request to correspondence author.

## References

[1] Stanford K. Introduction to the special issue "molecular basis and the pathogenesis of Enterohemorrhagic Escherichia coli infections". Toxins. 2020;12(12). 10.3390/toxins12120763. 10.3390/toxins12120763 PMC7761636 33287118

[2] Aslam N, Khan SU, Usman T, Ali T (2021). Phylogenetic genotyping, virulence genes and antimicrobial susceptibility of Escherichia coli isolates from cases of bovine mastitis. J Dairy Res.

[3] Awadallah MA, Ahmed HA, Merwad AM, Selim MA (2016). Occurrence, genotyping, Shiga toxin genes and associated risk factors of E. coli isolated from dairy farms, handlers and milk consumers. Vet J.

[4] Johnson JR, Russo TA. Molecular epidemiology of Extraintestinal pathogenic Escherichia coli. EcoSal Plus. 2018;8(1). 10.1128/ecosalplus.ESP-0004-2017. 10.1128/ecosalplus.esp-0004-2017 PMC11575673 29667573

[5] Gomes TA, Elias WP, Scaletsky IC, Guth BE, Rodrigues JF, Piazza RM (2016). Diarrheagenic Escherichia coli. Braz J Microbiol.

[6] Ntuli V, Njage PMK, Buys EM (2016). Characterization of Escherichia coli and other Enterobacteriaceae in producer-distributor bulk milk. J Dairy Sci.

[7] Howell AK, Tongue SC, Currie C, Evans J, Williams DJL, McNeilly TN (2018). Co-infection with Fasciola hepatica may increase the risk of Escherichia coli O157 shedding in British cattle destined for the food chain. Prev Vet Med.

[8] Ripa M, Galli L, Poli A, Oltolini C, Spagnuolo V, Mastrangelo A (2021). Secondary infections in patients hospitalized with COVID-19: incidence and predictive factors. Clin Microbiol Infect.

[9] Di Blasio A, Traversa A, Giacometti F, Chiesa F, Piva S, Decastelli L (2019). Isolation of Arcobacter species and other neglected opportunistic agents from aborted bovine and caprine fetuses. BMC Vet Res.

[10] Kolenda R, Burdukiewicz M, Schierack P (2015). A systematic review and meta-analysis of the epidemiology of pathogenic Escherichia coli of calves and the role of calves as reservoirs for human pathogenic E. coli. Front Cell Infect Microbiol.

[11] Denamur E, Clermont O, Bonacorsi S, Gordon D (2021). The population genetics of pathogenic Escherichia coli. Nat Rev Microbiol.

[12] Chapman TA, Wu XY, Barchia I, Bettelheim KA, Driesen S, Trott D (2006). Comparison of virulence gene profiles of Escherichia coli strains isolated from healthy and diarrheic swine. Appl Environ Microbiol.

[13] Robins-Browne RM, Holt KE, Ingle DJ, Hocking DM, Yang J, Tauschek M (2016). Are Escherichia coli Pathotypes still relevant in the era of whole-genome sequencing?. Front Cell Infect Microbiol.

[14] Tennant SM, Tauschek M, Azzopardi K, Bigham A, Bennett-Wood V, Hartland EL (2009). Characterisation of atypical enteropathogenic E. coli strains of clinical origin. BMC Microbiol.

[15] D L. (2000). Virulence factors of Escherichia coli O157 and other Shiga toxin-producing E. coli. J Appl Microbiol.

[16] Welch RA, Dellinger EP, Minshew B, Falkow S (1981). Haemolysin contributes to virulence of extra-intestinal E. coli infections. Nature.

[17] Robinson NB, Krieger K, Khan FM, Huffman W, Chang M, Naik A (2019). The current state of animal models in research: a review. Int J Surg.

[18] Garattini S, Grignaschi G (2017). Animal testing is still the best way to find new treatments for patients. Eur J Intern Med.

[19] Balls M (2018). Why are validated alternatives not being used to replace animal tests. Altern Lab Anim.

[20] Shen P, Yue Y, Park Y (2018). A living model for obesity and aging research: Caenorhabditis elegans. Crit Rev Food Sci Nutr.

[21] Kumar A, Baruah A, Tomioka M, Iino Y, Kalita MC, Khan M (2020). Caenorhabditis elegans: a model to understand host-microbe interactions. Cell Mol Life Sci.

[22] Iliff AJ, Xu XZS (2020). Elegans: a sensible model for sensory biology. J Neurogenet.

[23] Madende M, Albertyn J, Sebolai O, Pohl CH (2020). Caenorhabditis elegans as a model animal for investigating fungal pathogenesis. Med Microbiol Immunol.

[24] Park HH, Jung Y, Lee SV (2017). Survival assays using Caenorhabditis elegans. Mol Cells.

[25] Martineau CN, Kirienko NV, Pujol N (2021). Innate immunity in C. elegans. Curr Top Dev Biol.

[26] Darby CCC, Thomas JH, Manoil C (1999). Lethal paralysis of Caenorhabditis elegans by Pseudomonas aeruginosa. Proc Natl Acad Sci U S A.

[27] Urrutia A, Garcia-Angulo VA, Fuentes A, Caneo M, Legue M, Urquiza S (2020). Bacterially produced metabolites protect C. elegans neurons from degeneration. PLoS Biol.

[28] Ahamefule CS, Qin Q, Odiba AS, Li S, Moneke AN, Ogbonna JC (2020). Caenorhabditis elegans-based aspergillus fumigatus infection model for evaluating pathogenicity and drug efficacy. Front Cell Infect Microbiol.

[29] Dahan D, Preston GM, Sealey J, King KC (2020). Impacts of a novel defensive symbiosis on the nematode host microbiome. BMC Microbiol.

[30] Lee SH, Omi S, Thakur N, Taffoni C, Belougne J, Engelmann I (2018). Modulatory upregulation of an insulin peptide gene by different pathogens in C. elegans. Virulence.

[31] Huang X, Pan W, Kim W, White A, Li S, Li H (2020). Caenorhabditis elegans mounts a p38 MAPK pathway-mediated defence to Cutibacterium acnes infection. Cell Microbiol.

[32] Kim DH, Ewbank JJ (2018). Signaling in the innate immune response. WormBook.

[33] Dierking K, Yang W, Schulenburg H. Antimicrobial effectors in the nematode Caenorhabditis elegans: an outgroup to the Arthropoda. Philos Trans R Soc Lond Ser B Biol Sci. 2016;371(1695). 10.1098/rstb.2015.0299. 10.1098/rstb.2015.0299 PMC4874396 27160601

[34] Mir DA, Balamurugan K (2019). In vitro and in vivo efficacy of Caenorhabditis elegans recombinant antimicrobial protein against gram-negative bacteria. Biofouling.

[35] Iacob S, Iacob DG (2019). Infectious threats, the intestinal barrier, and its Trojan horse: Dysbiosis. Front Microbiol.

[36] Parvej MS, Alam MA, Shono M, Zahan MN, Masuma Parvez MM, Ansari WK (2020). Prevalence of virulence genes of Diarrheagenic Escherichia coli in fecal samples obtained from cattle, poultry and diarrheic patients in Bangladesh. Jpn J Infect Dis.

[37] Crofts AA, Giovanetti SM, Rubin EJ, Poly FM, Gutierrez RL, Talaat KR (2018). Enterotoxigenic E. coli virulence gene regulation in human infections. Proc Natl Acad Sci U S A.

[38] Geue L, Menge C, Eichhorn I, Semmler T, Wieler LH, Pickard D (2017). Evidence for contemporary switching of the O-antigen gene cluster between Shiga toxin-producing Escherichia coli strains colonizing cattle. Front Microbiol.

[39] Liu B, Furevi A, Perepelov AV, Guo X, Cao H, Wang Q (2020). Structure and genetics of Escherichia coli O antigens. FEMS Microbiol Rev.

[40] DebRoy C, Fratamico PM, Roberts E (2018). Molecular serogrouping of Escherichia coli. Anim Health Res Rev.

[41] Backes C, Martinez-Martinez D, Cabreiro F (2021). C. elegans: a biosensor for host-microbe interactions. Lab Anim.

[42] Foster KR, Schluter J, Coyte KZ, Rakoff-Nahoum S (2017). The evolution of the host microbiome as an ecosystem on a leash. Nature.

[43] Leung MC, Williams PL, Benedetto A, Au C, Helmcke KJ, Aschner M (2008). Caenorhabditis elegans: an emerging model in biomedical and environmental toxicology. Toxicol Sci.

[44] Apfeld J, Alper S (2018). What can we learn about human disease from the Nematode C. elegans?. Methods Mol Biol.

[45] Morch MGM, Moller KV, Hesselager MO, Harders RH, Kidmose CL, Buhl T (2021). The TGF-beta ligand DBL-1 is a key player in a multifaceted probiotic protection against MRSA in C. elegans. Sci Rep.

[46] Gumienny TL, Savage-Dunn C. TGF-beta signaling in C. elegans. WormBook. 2013:1–34. 10.1895/wormbook.1.22.2. 10.1895/wormbook.1.22.2 PMC5081272 23908056

[47] Lakdawala MFMB, Faure L, Vora M, Padgett RW, Gumienny TL (2019). Genetic interactions between the DBL-1_BMP-like pathway and dpy body size-associated genes in Caenorhabditis elegans. Mol Biol Cell.

[48] Pereira AG, Gracida X, Kagias K, Zhang Y (2020). C. elegans aversive olfactory learning generates diverse intergenerational effects. J Neurogenet.

[49] Liu H, Zhang Y (2020). What can a worm learn in a bacteria-rich habitat?. J Neurogenet.

[50] Mallo GVKC, Couillault C, Pujol N, Granjeaud S, Kohara Y, Ewbank JJ (2002). Inducible antibacterial defense system in C. elegans. Curr Biol.

[51] Zhang X, Zhang Y (2012). DBL-1, a TGF-beta, is essential for Caenorhabditis elegans aversive olfactory learning. Proc Natl Acad Sci U S A.

[52] Kwon G, Lee J, Koh JH, Lim YH (2018). Lifespan extension of Caenorhabditis elegans by Butyricicoccus pullicaecorum and Megasphaera elsdenii with probiotic potential. Curr Microbiol.

[53] Kim DH, Feinbaum R, Alloing G, Emerson FE, Garsin DA, Inoue H, Tanaka-Hino M, Hisamoto N, Matsumoto K, Tan M-W, Ausubel FM (2002). A conserved p38 MAP kinase pathway in Caenorhabditis elegans innate immunity. Science.

[54] Bolz DD, Tenor JL, Aballay A (2010). A conserved PMK-1/p38 MAPK is required in caenorhabditis elegans tissue-specific immune response to Yersinia pestis infection. J Biol Chem.

[55] Tissenbaum HA (2018). DAF-16: FOXO in the context of C. elegans. Curr Top Dev Biol.

[56] Radek K, Gallo R (2007). Antimicrobial peptides: natural effectors of the innate immune system. Semin Immunopathol.

[57] Alegado RA, Tan MW (2008). Resistance to antimicrobial peptides contributes to persistence of Salmonella typhimurium in the C. elegans intestine. Cell Microbiol.

[58] Froy O (2005). Convergent evolution of invertebrate defensins and nematode antibacterial factors. Trends Microbiol.

