# Supplementary material for: Immunological pathogenesis of Bovine E. coli infection in a model of C. elegans
Source: BMC Microbiol. 2022 Dec 20;22:311. doi: 10.1186/s12866-022-02733-5 (PMC9764636; doi:10.1186/s12866-022-02733-5)
Supplement: Supplementary file 1 — Additional file 1. [file 12866_2022_2733_MOESM1_ESM.pdf]

Immunological Pathogenesis of Bovine *E. coli* Infection in a Model of *C. elegans*

Hao Peng[1†], Huili Bai[1†], Yan Pan[2†], Jun Li[1]*, Zhe Pei[3], Yuying Liao[1], Cuilan Wu[1], Changting Li[1], Li Tao[1], Shuhong Zhong[1], Chunxia Ma[1], Zhongwei Chen[1], Xiaoning Li[1], Yu Gong[4], Leping Wang[1], Fengsheng Li[1]

*Correspondence:

Prof. Jun Li, Guangxi Key Laboratory of Veterinary Biotechnology, Guangxi Veterinary Research Institute, Nanning 530001, China

Tel: 86-0771-3186349

E-mail: jlee9981@163.com

† These authors contribute equally to this paper.

[1]Guangxi Key Laboratory of Veterinary Biotechnology, Guangxi Veterinary Research Institute, Nanning China; [2]Guangxi Agricultural Vocational University, Nanning, China; [3]The City College of New York, New York, USA and [4]Animal Science and Technology Station of Guizhou, Guiyang, China.

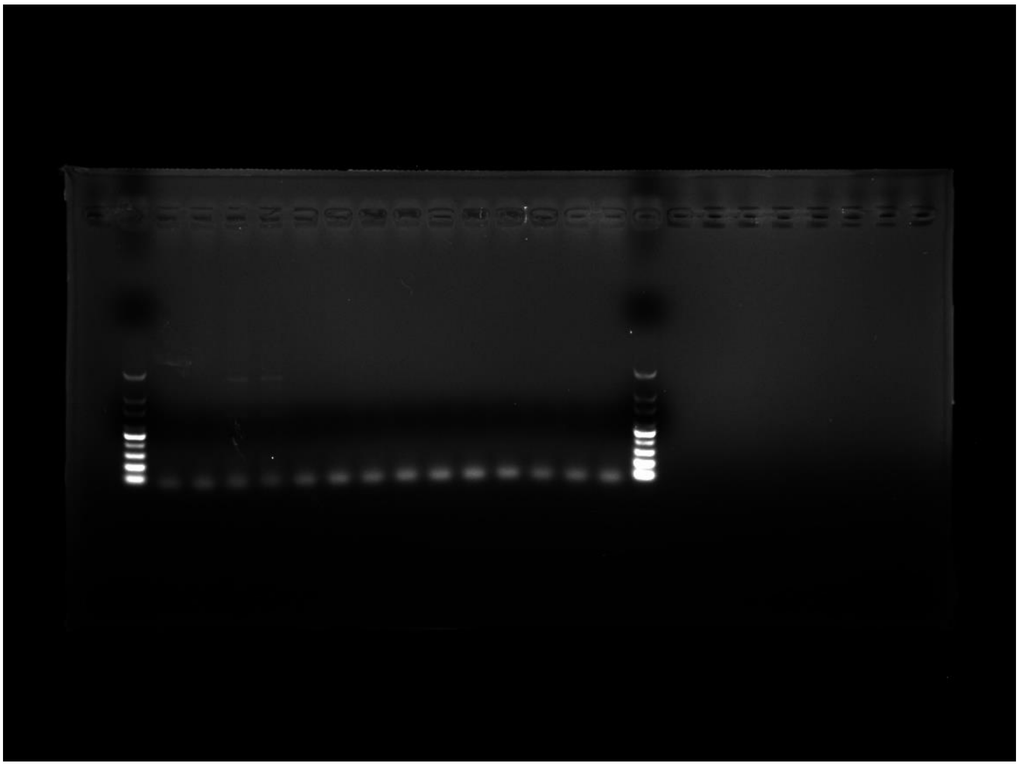

Fig. S1 Amplification results of virulence factor 987P

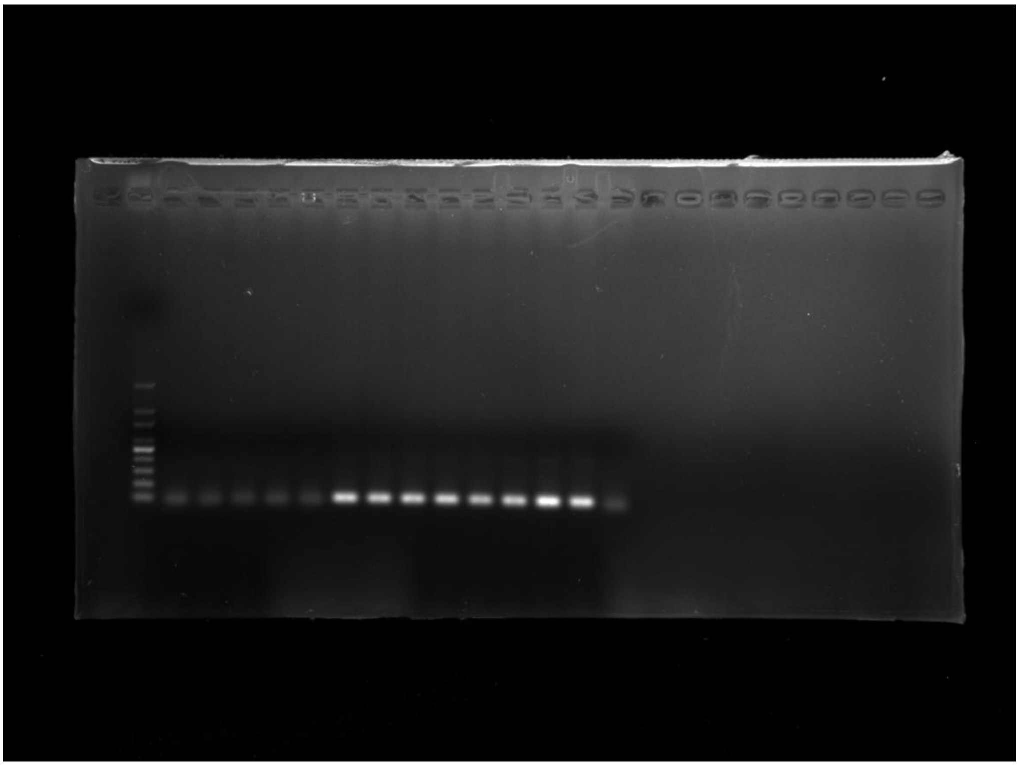

Fig. S2 Amplification results of virulence factor astA

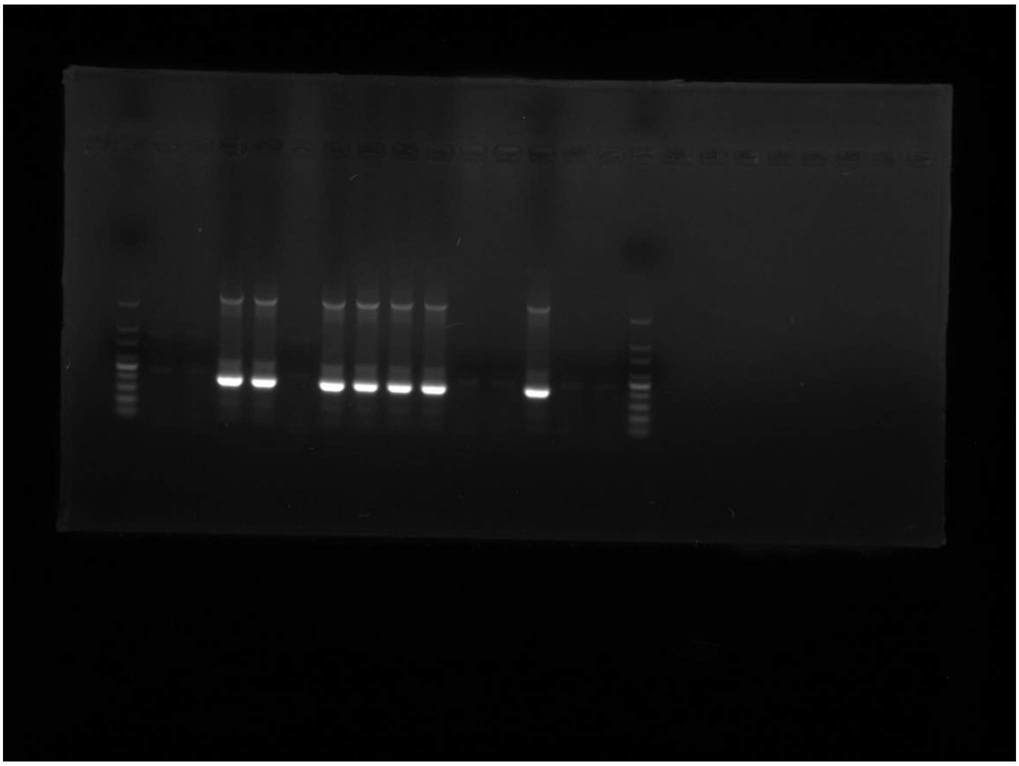

Fig. S3 Amplification results of virulence factor CS31

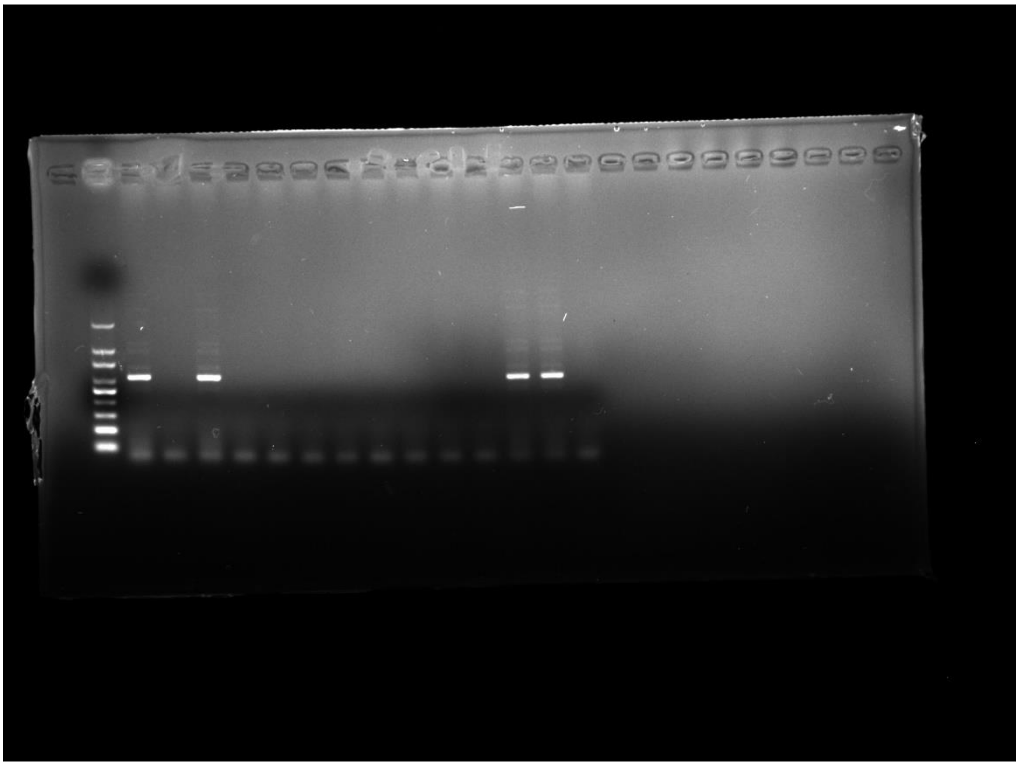

Fig. S4 Amplification results of virulence factor eaeA

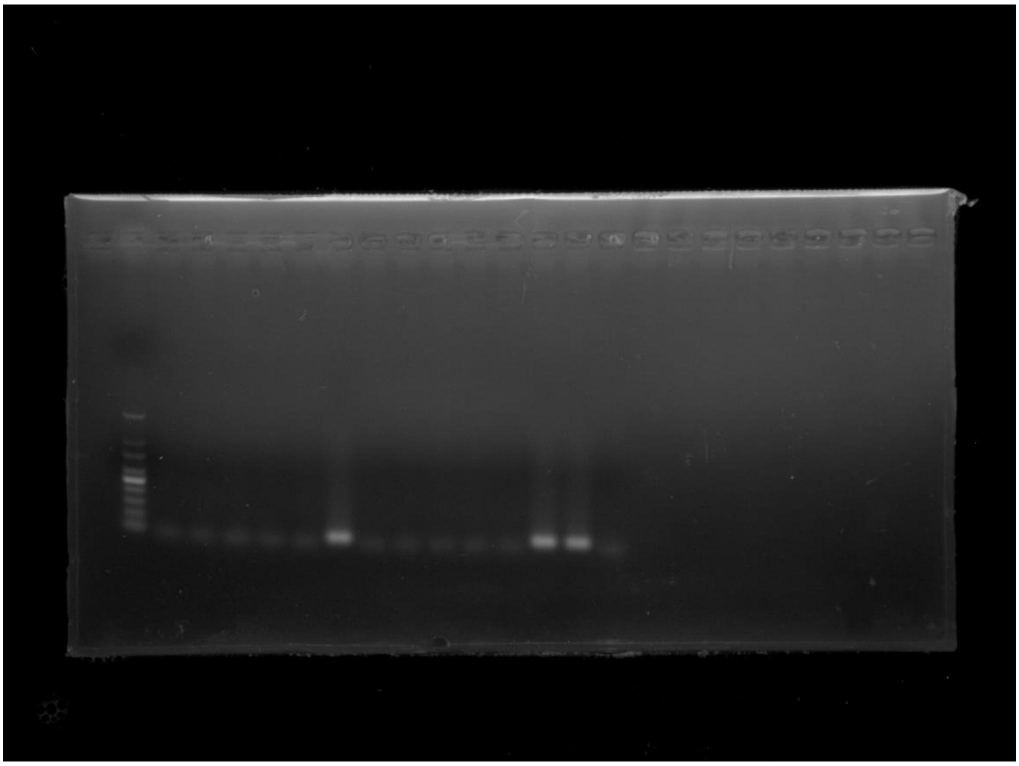

Fig. S5 Amplification results of virulence factor EAST1

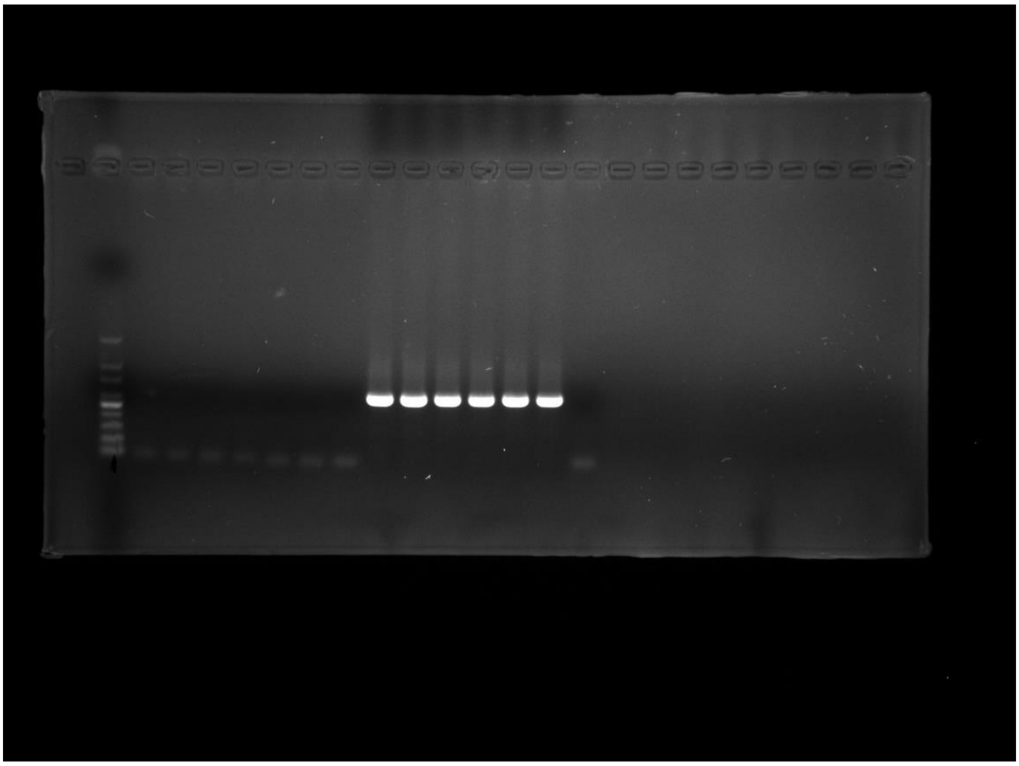

Fig. S6 Amplification results of virulence factor ent

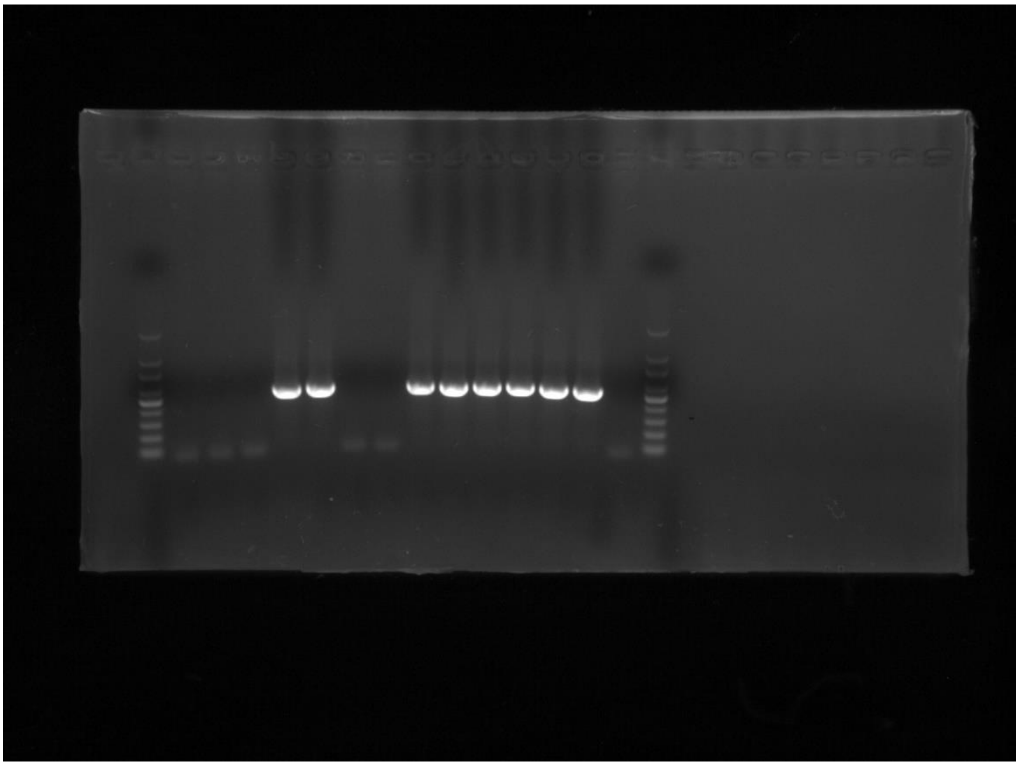

Fig. S7 Amplification results of virulence factor escV

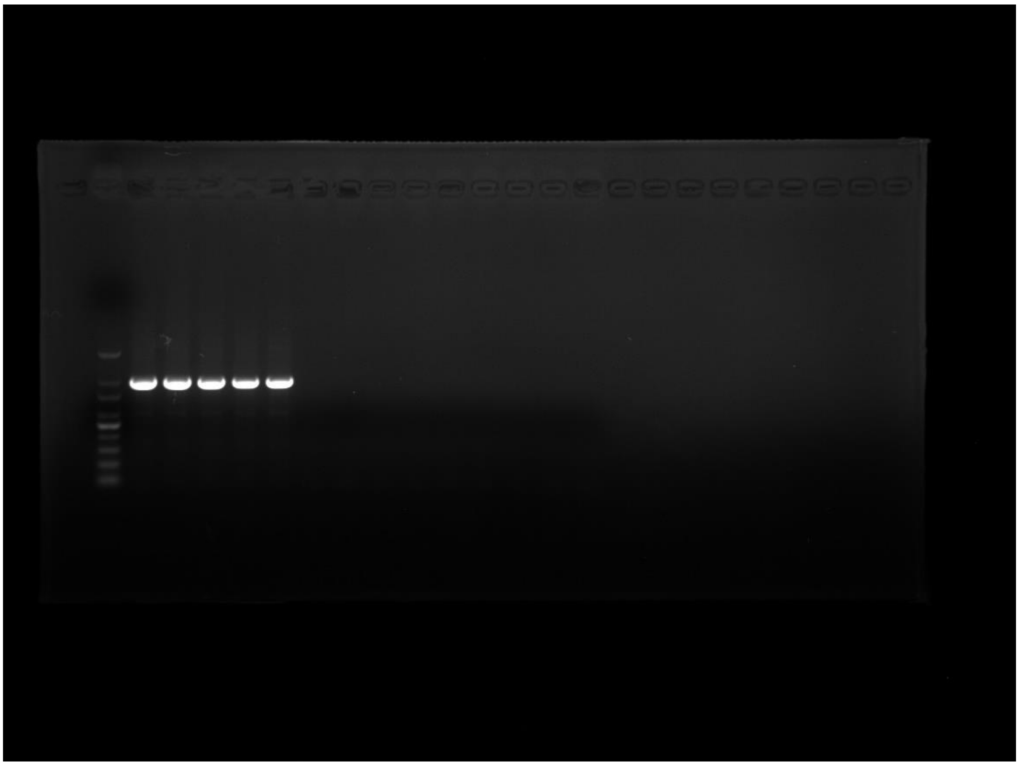

Fig. S8 Amplification results of virulence factor FyuA

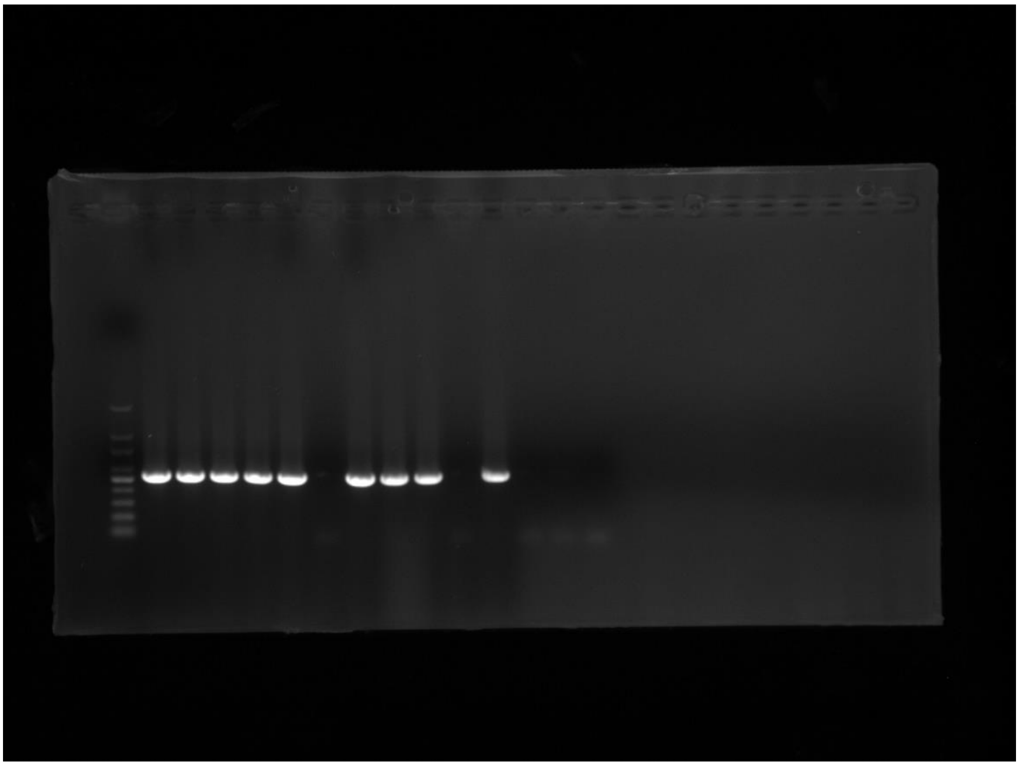

Fig. S9 Amplification results of virulence factor h1yA

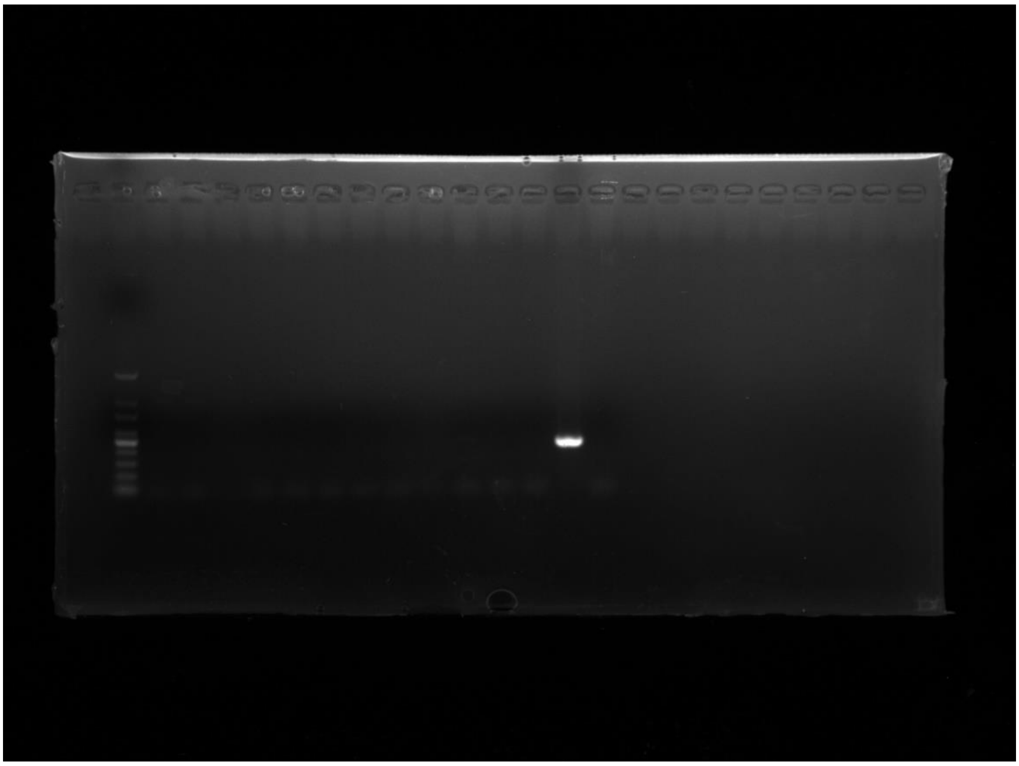

Fig. S10 Amplification results of virulence factor ipaH

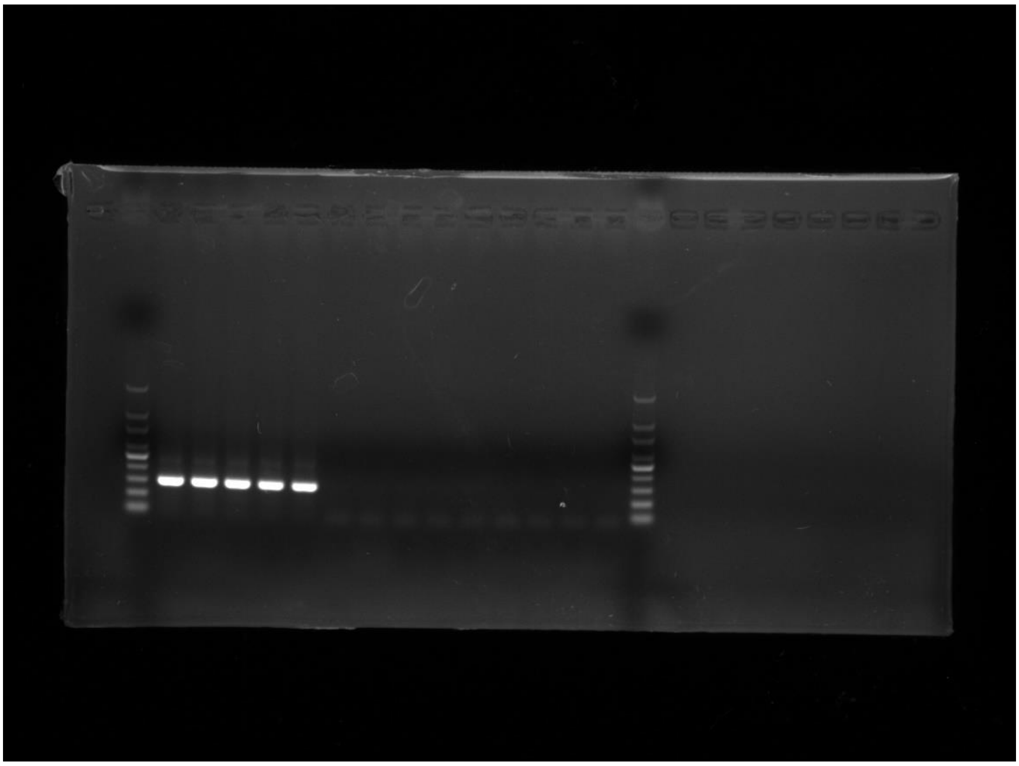

Fig. S11 Amplification results of virulence factor irp2

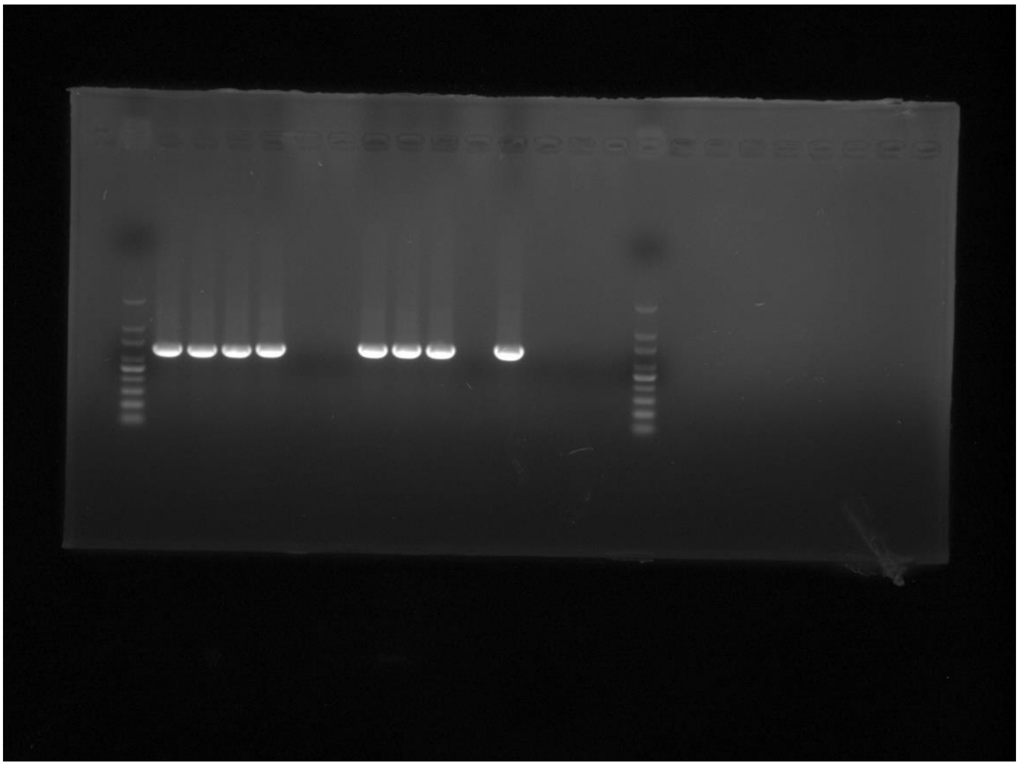

Fig. S12 Amplification results of virulence factor iucD

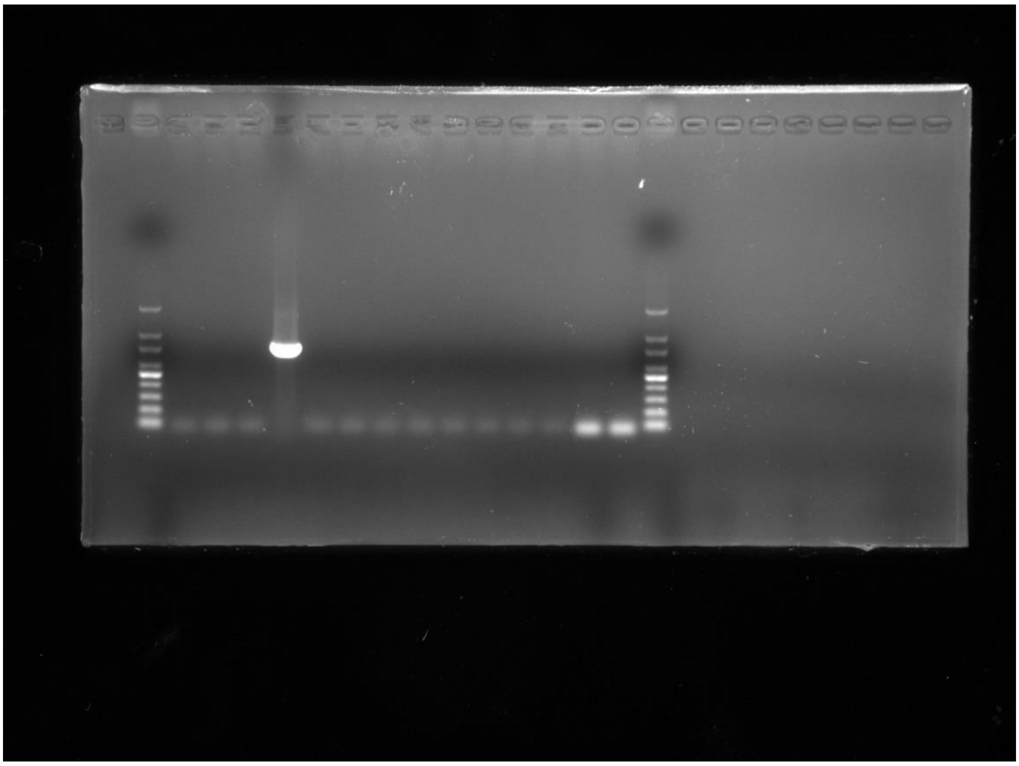

Fig. S13 Amplification results of virulence factor K88

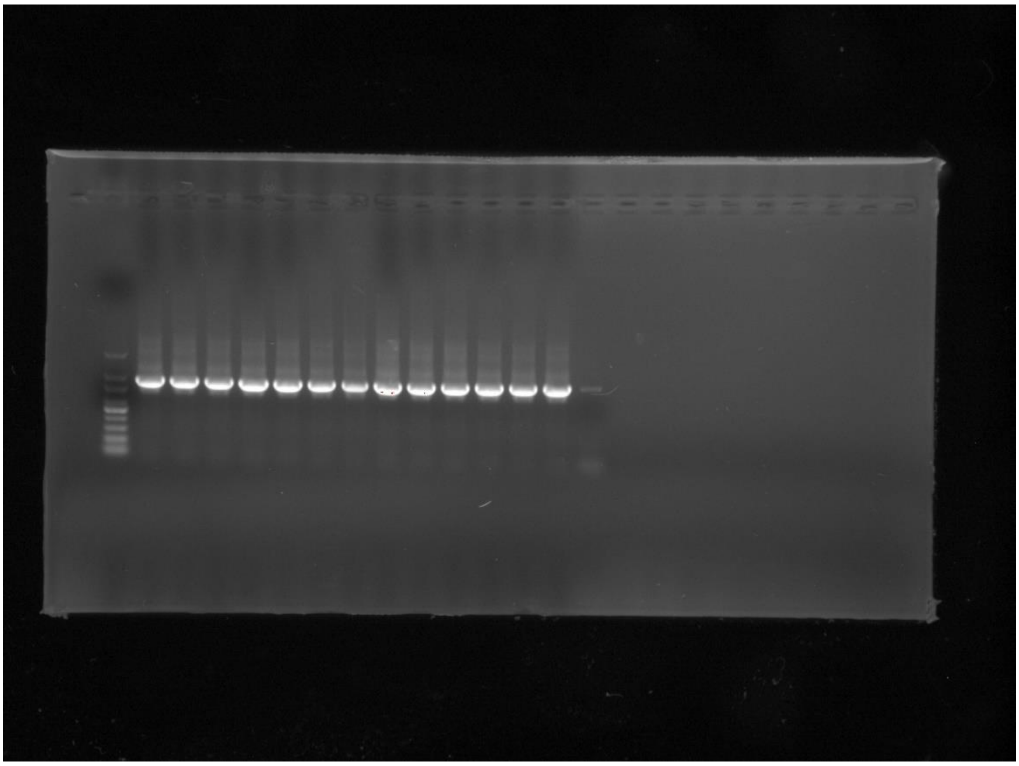

Fig. S14 Amplification results of virulence factor ompA

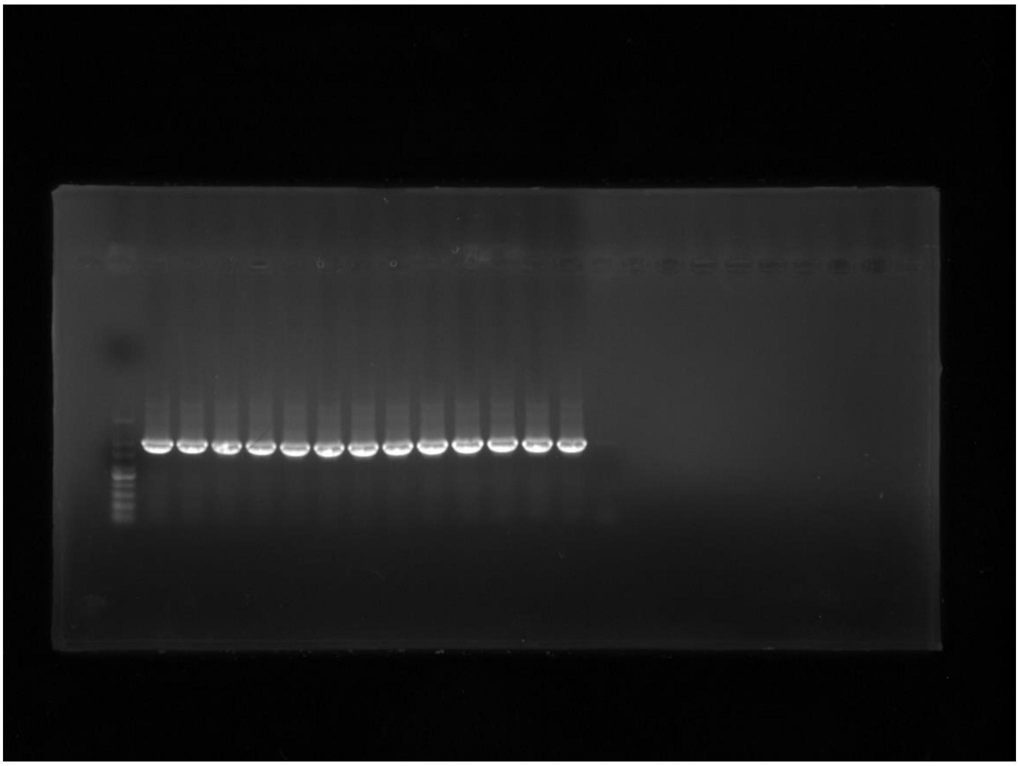

Fig. S15 Amplification results of virulence factor phoA

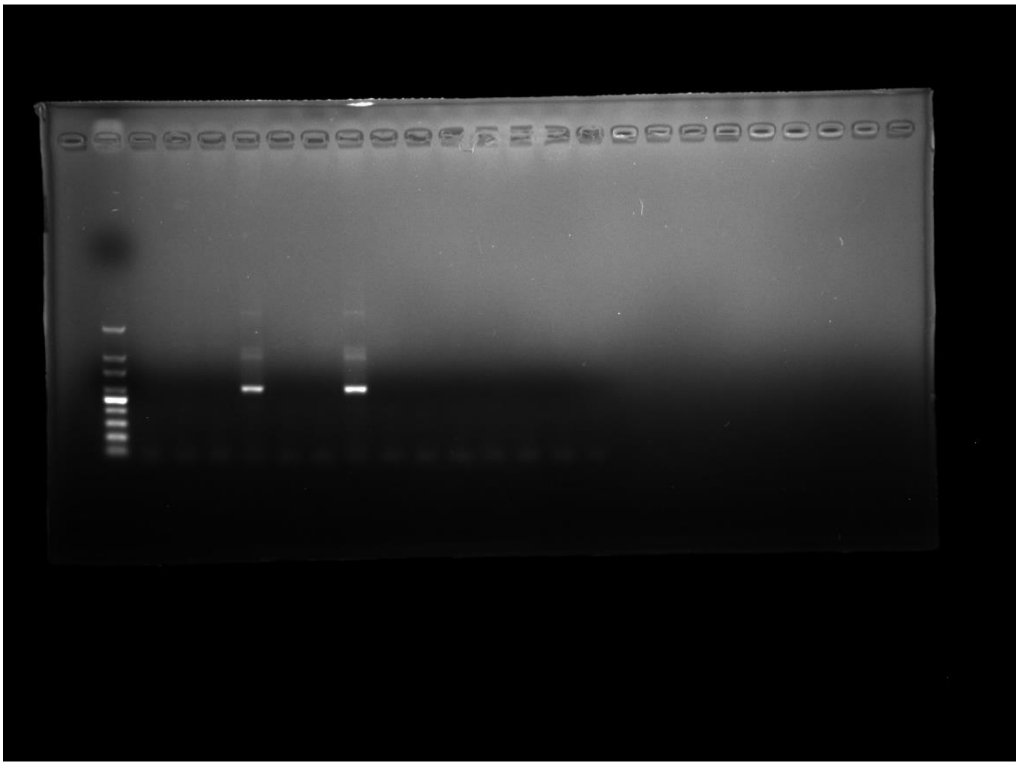

Fig. S16 Amplification results of virulence factor SepA

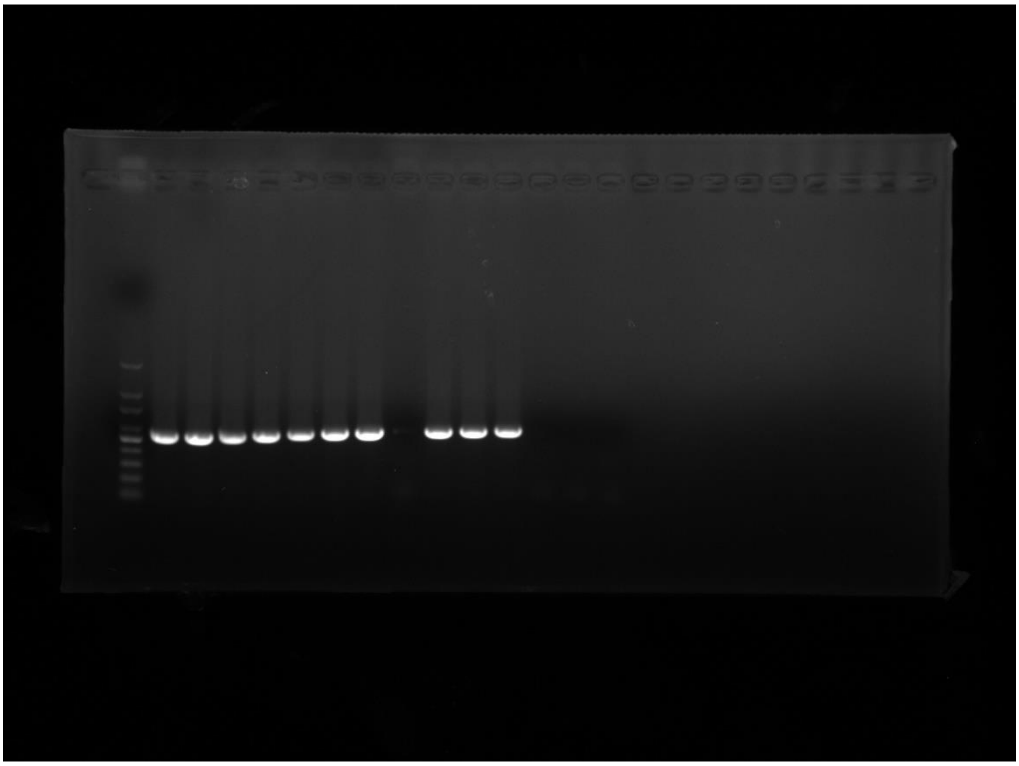

Fig. S17 Amplification results of virulence factor Stx1

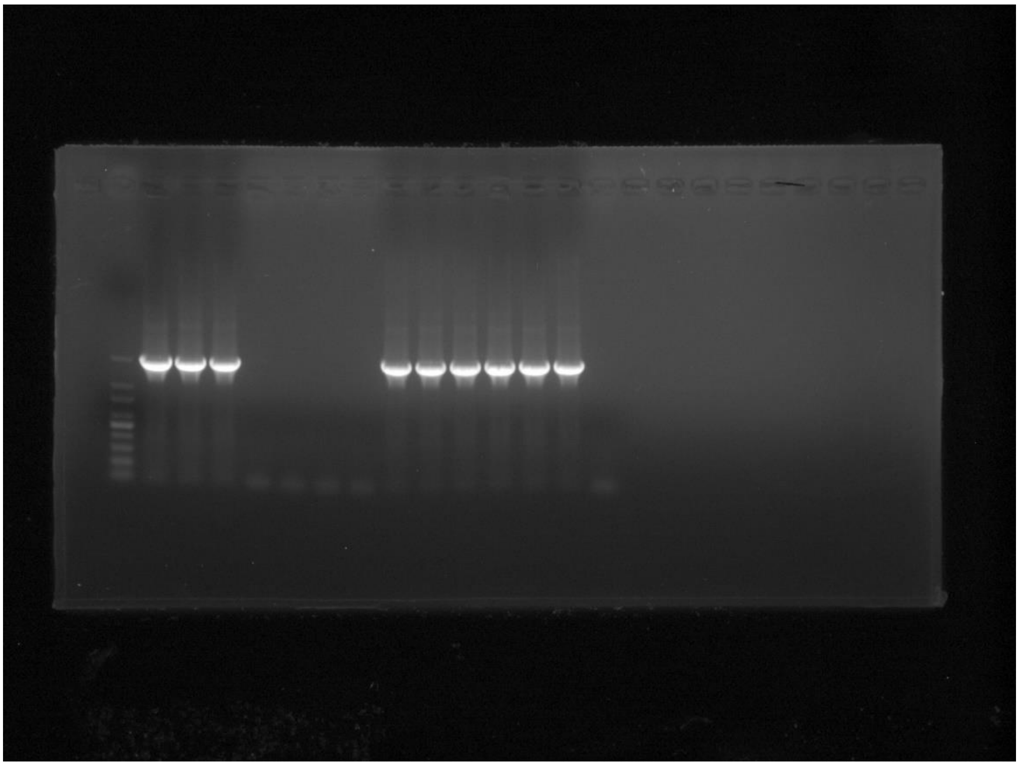

Fig. S18 Amplification results of virulence factor uidA

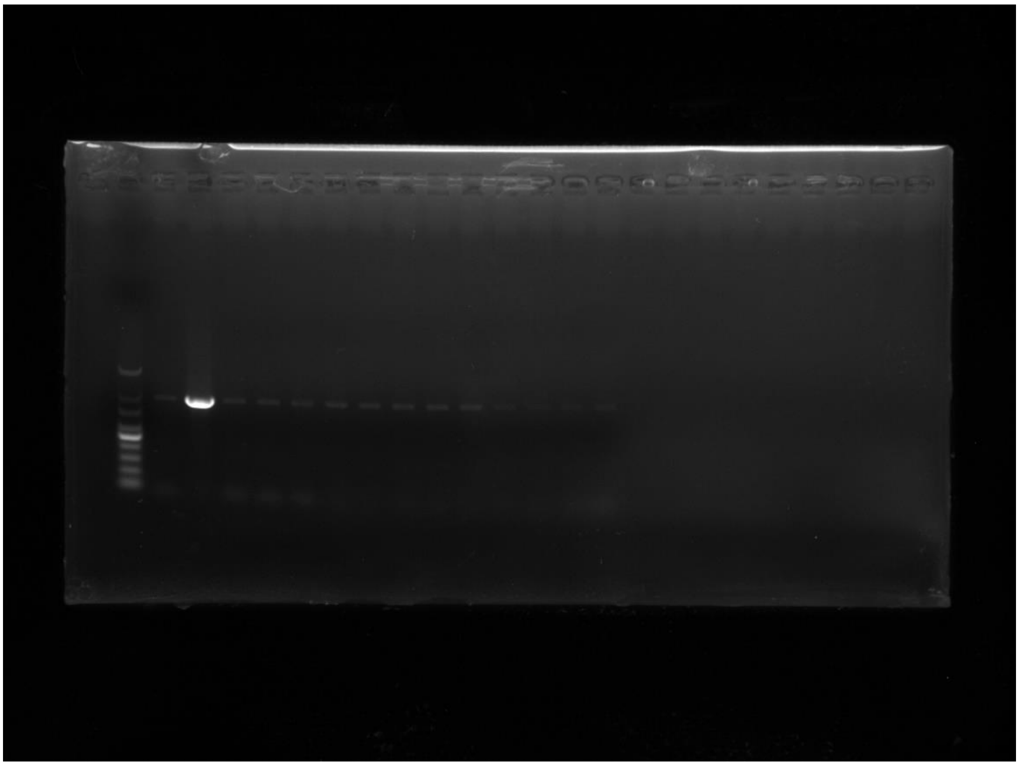

Fig. S19 Amplification results of virulence factor vat
