# Supplementary material for: Immunological pathogenesis of Bovine E. coli infection in a model of C. elegans
Source: BMC Microbiol. 2022 Dec 20;22:311. doi: 10.1186/s12866-022-02733-5 (PMC9764636; doi:10.1186/s12866-022-02733-5)
Supplement: Supplementary file 2 — Additional file 2. [file 12866_2022_2733_MOESM2_ESM.pdf]

Immunological Pathogenesis of Bovine *E. coli* Infection in a Model of *C. elegans*

Hao Peng[1†], Huili Bai[1†], Yan Pan[2†], Jun Li[1]*, Zhe Pei[3], Yuying Liao[1], Cuilan Wu[1], Changting Li[1], Li Tao[1], Shuhong Zhong[1], Chunxia Ma[1], Zhongwei Chen[1], Xiaoning Li[1], Yu Gong[4], Leping Wang[1], Fengsheng Li[1]

*Correspondence:

Prof. Jun Li, Guangxi Key Laboratory of Veterinary Biotechnology, Guangxi Veterinary Research Institute, Nanning 530001, China

Tel: 86-0771-3186349

E-mail: jlee9981@163.com

† These authors contribute equally to this paper.

[1]Guangxi Key Laboratory of Veterinary Biotechnology, Guangxi Veterinary Research Institute, Nanning China; [2]Guangxi Agricultural Vocational University, Nanning, China; [3]The City College of New York, New York, USA and [4]Animal Science and Technology Station of Guizhou, Guiyang, China.

To clone the set of virulence factors, this study synthesized all primers according to previous studies (Table S1 A, B).

Table S1（A） Primers for *E.coli* virulence factors

Gene name	Primer sequences（5’-3’）	Length of target fragments /bp	Annealing temperature /℃
irp2	F:AAGGATTCGCTGTTACCGGA<br>R:TCGGCCAGGATGATTCGTCG	301	60
Fyu A	F:ACACGGCTTATCCTCTGGC<br>R:GGCATCTTGACGATTAACGAA	953	58
F41	F:GAGGGACTTTCATCTTTTAG<br>R:AGTCCATTCCATTTATAGGC	431	58
K88	F:GATGAAAAAGACTCTGATTGCA<br>R:GATTGCTACGTTCAGCGGAGCG	841	54
K99	F:CTGAAAAAAACACTGCTAGCTATT<br>R:CATATAAGTGACTAAGAAGGATGC	314	52.5
987P	F:GTTACTGCCAGTCTATGCCAAGTG<br>R:TCGGTGTACCTGCTGAACGAATAG	463	56
F17	F:GGGCTGACAGAGGAGGTGGGGC<br>R:CCCGGCGACAACTTCATCACCGG	411	61
F18	F:GTGAAAAGACTAGTGTTTATTTC<br>R:CTTGTAAGTAACCGCGTAAGC	510	55
Stx1	F:TTAGACTTCTCGACTGCAAAG<br>R:TGTTGTACGAAATCCCCTCTG	531	52
Stx2	F:CCATGACAACGGACAGCAGTT<br>R:CCTGTCAACTGAGCAGCACTTTG	779	58.3
STb	F:TGCC TATGCA TCT ACACAATC<br>R:GC AG TGAGA AATGGAC AATG	283	TD
STa	F:TCCCCTCTTTTAGTCAGTCAACTG<br>R:GCACAGGCAGGATTACAACAAAGT	163	56
LT	F:GGCGACAGATTATACCGTGC<br>R:CGGTCTCTATATTCCCTGTT	450	54
eae A	F:ATTACTGAGATTAAGGCTGAT<br>R:ATTTATTTGCAGCCCCCCAT	682	57
hly A	F:GCATCATCAAGCGTACGTTCC<br>R:AATGAGCCAAGCTGGTTAAGCT	534	60
SepA	F:TAAAAC-CCGCCGCCTGAGTA<br>R:TGCCGGTGAACAGGAGGTTT	611	62
escV	F:ATTCTGGCTCTCTTCTTCTTTATGGCTG<br>R:CGTCCCCTTTTACAAACTTCATCGC	544	63
ent	F:TGGGCTAAAAGAAGACACACTG<br>R:CAAGCATCCTUATTATCTCACC	629	63
bfpA	F:GGAAGTCAAATTCATGGGGGTAT<br>R:GGAATCAGACGCAGACTGGTAGT	326	55
bfpB	F:GACACCTCATTGCTGAAGTCG<br>R:CCAGAACACCTCCGTTATGC	910	63
ipaH	F:GAAAACCCTCCTGGTCCA TCAGG<br>R:GCCGGTCAGGCACCCTCTGAGAGTAC	437	63
invE	F:CGATAGATGGCGAGAAACTGGCGAGA TTATATCCCG<br>R:CGATCAAGAATCCCTAACAGAAGAATCAC	766	63

Table S1（B） Primers for cloning *E.coli* virulence factors

Gene name	Primer sequences（5’-3’）	The length of target fragments /bp	Annealing temperature /°C
aggR	F:ACGCAGAGTTGCCTGATAAAG<br>R:AATACAGAATCGTCATCAGC	400	63
pic	F:AGCCGTTTCCGCAGAAGCC<br>R:AAATGTCAGTGAACCGACGATTGG	1111	63
astA	F:TGCCATCAACACAGTATATCCG<br>R:ACGGCTTTGTAGTCCTTCCA T	102	63
STIa	F:CCTCTTTTAGYCAGACARCTGAATCASTTG<br>R:CAGGCAGGATTACAACAAAGTTCACAG	157	63
STI	F:TGTCTTTTTCACCTTTCGCTC<br>R:CGGTACAAGCAGGATTACAACAC	171	63
uidA	F:ATGCCAGTCCAGCGTTTTTGC<br>R:AAAGTGTGGGTCAAT AATCAGGAAGTG	1487	63
afa/draB	F:TAAGGAAGTGAAGGAGCGTG<br>R:CCAGTAACTGTCCGTGACA	810	58
iha	F:TAGTGCGTTGGGTTATCGCTC<br>R:AAGCCAGAGTGGTTATTCGC	609	58
sfa/foCD	F:GTCCTGACTCATCTGAAACTGCA<br>R:CGGAGAACTGGGTGCATCTTA	1242	59
sitDep.a	F:TTGAGAACGACAGCGACTTC<br>R:CTATCGAGCAGGTGAGGA	1052	60
sitD chr.	F:ACTCCCATACACAGGATCTG<br>R:CTGTCTGTGTCCGGAATGA	554	58
iucD.a	F:ACAAAAAGTTCTATCGCTTCC<br>R:CCTGATCCAGATGATGCTC	714	57
neuC	F:GGTGGTACATTCCGGGATGTC<br>R:AGGTGAAAAGCCTGGTAGTGTG	676	57
ompA	F:AGCTATCGCGATTGCAGTG<br>R:GGTGTTGCCAGTAACCGG	919	57.5
sat	F:TGCTGGCTCTGGAGGAAC<br>R:TTGAACATTCAGAGTACCGGG	667	57
vat	F:TCCTGGGACATAATGGCTAG<br>R:GTGTCAGAACGGAATTGTC	981	58
phoA	F:GCAAACCGGACTACGTCACC<br>R:TGAATCCTCTTCGGAGTTCCC	947	57
CS31A	F:GGGCGCTCTCTGCTTCAAC<br>R:CGCCCTAATTGCTGGCGAC	402	56
EAST1	F:ATGCCATCAACACAGTATATC<br>R:TCAGGTCGCGAGTGACGG	117	55
