# Supplementary material for: Immunological pathogenesis of Bovine E. coli infection in a model of C. elegans
Source: BMC Microbiol. 2022 Dec 20;22:311. doi: 10.1186/s12866-022-02733-5 (PMC9764636; doi:10.1186/s12866-022-02733-5)
Supplement: Supplementary file 3 — Additional file 3. [file 12866_2022_2733_MOESM3_ESM.pdf]

Immunological Pathogenesis of Bovine *E. coli* Infection in a Model of *C. elegans*

Hao Peng[1†], Huili Bai[1†], Yan Pan[2†], Jun Li[1]*, Zhe Pei[3]， Yuying Liao[1], Cuilan Wu[1], Changting Li[1], Li Tao[1], Shuhong Zhong[1], Chunxia Ma[1], Zhongwei Chen[1], Xiaoning Li[1], Yu Gong[4], Leping Wang[1], Fengsheng Li[1]

*Correspondence:

Prof. Jun Li, Guangxi Key Laboratory of Veterinary Biotechnology, Guangxi Veterinary Research Institute, Nanning 530001, China

Tel: 86-0771-3186349

E-mail: jlee9981@163.com

† These authors contribute equally to this paper.

[1]Guangxi Key Laboratory of Veterinary Biotechnology, Guangxi Veterinary Research Institute, Nanning China; [2]Guangxi Agricultural Vocational University， Nanning, China; [3]The City College of New York, New York, USA and [4]Animal Science and Technology Station of Guizhou, Guiyang, China.

Primers used for synthesis immunity genes of *C. elegans*

Table S2 (A, B) summarized primers in this study to clone immune genes which was studied. All listed primers have been tested previously.

Table S2（A） Primers for immune gene cloning

Gene name	Primer sequence（5’-3’）	Target fragment /bp
Act-1	F:CCCCACTCAATCCAAAGGCT	121
	R:GTACGTCCGGAAGCGTAGAG	
Dbl-1	F:TTTTGCGGCGAACAAATCGT	194
	R:TTCGCTGTTGCCTGTTTGTG	
Tir-1	F:TTGGGTGCACAAAGAGCTGA	223
	R:GGTCGGTGTCGTTCTGTTCA	
Nsy-1	F:AGCGGCTCGATCAACAAGAA	122
	R:CCCATTCCACCGATATGCGA	
Sek-1	F:CACTGTTTGGCGACGATGAG	158
	R:ATTCCGTCCACGTTGCTGAT	
Pmk-1	F:CCAAAAATGACTCGCCGTGA	115
	R:CTTTTGCAGTTGGACGACGA	
Skn-1	F:CTGGCATCCTCTACCACCAC	153
	R:TTGGTGATGATGGCCGTGTT	
Daf-16	F:TCGTCTCGTGTTTCTCCAGC	181
	R:TAATCGGCTTCGACTCCTGC	

Table S2 （B）Primers for immune gene cloning

Gene name	Primer sequence（5’-3’）	Target fragment /bp
Age-1	F:CTCCTGAACCGACTGCCAAT	359
	R:AAATGCGAGTTCGGAGAGCA	
Lys-7	F:GTACAGCGGTGGAGTCACTG	153
	R:GCCTTGAGCACATTTCCAGC	
Clec-60	F:CGGTTTCAATGCGGTATGGC	219
	R:TGAAGCTGTGGTTGAGGCAT	
Clec-85	F:CCAATGGGATGACGGAACCA	121
	R:CTTCTGTCCAGCCAACGTCT	
Abf-3	F:AACAGATTGGGGTCAGCTCG	189
	R:TGGAGACCATTATTGCCGGG	
Abf-2	F:CCGTTCCCTTTTCCTTGCAC	176
	R:GACGACCGCTTCGTTTCTTG	
Spp-1	F:TGGACTATGCTGTTGCCGTT	106
	R:ACGCCTTGTCTGGAGAATCC	
