# Supplementary material for: Immunological pathogenesis of Bovine E. coli infection in a model of C. elegans
Source: BMC Microbiol. 2022 Dec 20;22:311. doi: 10.1186/s12866-022-02733-5 (PMC9764636; doi:10.1186/s12866-022-02733-5)
Supplement: Supplementary file 6 — Additional file 6. [file 12866_2022_2733_MOESM6_ESM.pdf]

Immunological Pathogenesis of Bovine *E. coli* Infection in a Model of *C. elegans*

Hao Peng[1†], Huili Bai[1†], Yan Pan[2†], Jun Li[1]*, Zhe Pei[3], Yuying Liao[1], Cuilan Wu[1], Changting Li[1], Li Tao[1], Shuhong Zhong[1], Chunxia Ma[1], Zhongwei Chen[1], Xiaoning Li[1], Yu Gong[4], Leping Wang[1], Fengsheng Li[1]

*Correspondence:

Prof. Jun Li, Guangxi Key Laboratory of Veterinary Biotechnology, Guangxi Veterinary Research Institute, Nanning 530001, China

Tel: 86-0771-3186349

E-mail: jlee9981@163.com

† These authors contribute equally to this paper.

[1]Guangxi Key Laboratory of Veterinary Biotechnology, Guangxi Veterinary Research Institute, Nanning China; [2]Guangxi Agricultural Vocational University, Nanning, China; [3]The City College of New York, New York, USA and [4]Animal Science and Technology Station of Guizhou, Guiyang, China.

Table S4. Longest survival time and LT50 of *Caenorhabditis elegans* infected with 13 strains of bovine *Escherichia coli*

strain	Longest survival time of *Caenorhabditis elegans*	LT50
*E.coli*1	7d	3d
*E.coli*2	7d	3.5d
*E.coli*3	7d	3d
*E.coli*4	8d	3.8d
*E.coli*5	11d	6d
*E.coli*6	8d	3.2d
*E.coli*7	9d	4.5d
*E.coli*8	7d	3.75d
*E.coli*9	10d	4d
*E.coli*10	9d	3d
*E.coli*11	10d	4.3d
*E.coli*12	8d	3.3d
*E.coli*13	10d	5d
