# Supplementary material for: Immunological pathogenesis of Bovine E. coli infection in a model of C. elegans
Source: BMC Microbiol. 2022 Dec 20;22:311. doi: 10.1186/s12866-022-02733-5 (PMC9764636; doi:10.1186/s12866-022-02733-5)
Supplement: Supplementary file 7 — Additional file 7. [file 12866_2022_2733_MOESM7_ESM.pdf]

Immunological Pathogenesis of Bovine *E. coli* Infection in a Model of *C. elegans*

Hao Peng[1†], Huili Bai[1†], Yan Pan[2†], Jun Li[1]*, Zhe Pei[3], Yuying Liao[1], Cuilan Wu[1], Changting Li[1], Li Tao[1], Shuhong Zhong[1], Chunxia Ma[1], Zhongwei Chen[1], Xiaoning Li[1], Yu Gong[4], Leping Wang[1], Fengsheng Li[1]

*Correspondence:

Prof. Jun Li, Guangxi Key Laboratory of Veterinary Biotechnology, Guangxi Veterinary Research Institute, Nanning 530001, China

Tel: 86-0771-3186349

E-mail: jlee9981@163.com

† These authors contribute equally to this paper.

[1]Guangxi Key Laboratory of Veterinary Biotechnology, Guangxi Veterinary Research Institute, Nanning China; [2]Guangxi Agricultural Vocational University, Nanning, China; [3]The City College of New York, New York, USA and [4]Animal Science and Technology Station of Guizhou, Guiyang, China.

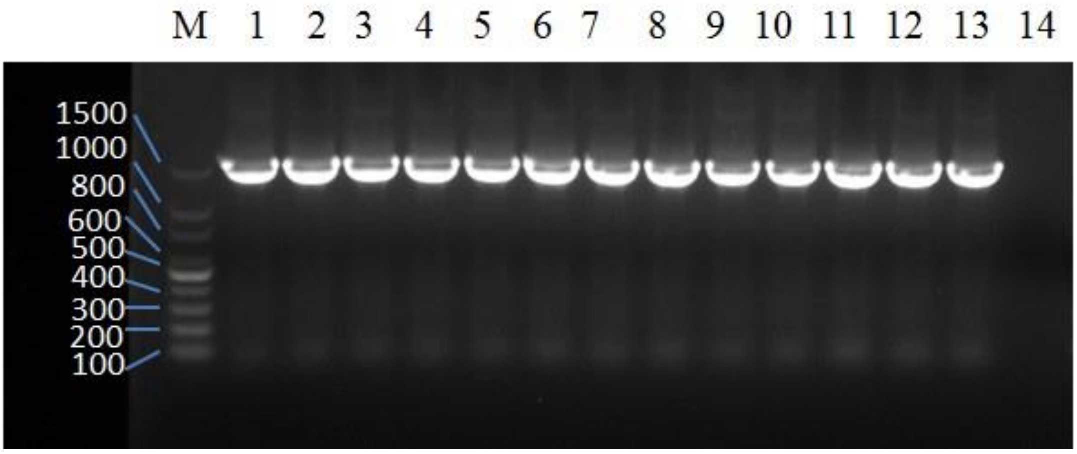

Figure S20. 16SrDNA PCR amplification results of 13 strains of bovine *Escherichia coli.* The blue lines represent the different bands of the marker

(M,marker.1-13, 13 strains of bovine Escherichia coli.14,control.)

Percent Identity

Divergence

	1	2	3	4	5	6	7	8	9	10	11	12	13	14	15	16	17	18	19	20	21		
1		99.3	99.4	99.3	99.9	99.7	99.6	99.3	99.2	97.3	99.2	99.6	99.4	100.0	99.2	98.5	99.3	99.1	100.0	100.0	100.0	1	GX11.seq
2	0.7		99.8	99.9	99.3	99.0	99.6	100.0	99.8	97.7	99.7	99.3	99.3	99.3	99.9	99.1	99.2	99.6	99.2	99.2	99.3	2	GX12.seq
3	0.6	0.2		99.7	99.3	99.1	98.5	98.9	99.6	99.6	99.3	99.3	99.2	99.3	99.7	98.3	99.1	98.9	99.0	99.3	99.4	3	GX13.seq
4	0.7	0.1	0.3		99.3	97.1	97.0	97.8	98.9	96.4	97.8	99.2	98.8	99.3	99.8	98.3	98.8	98.9	98.9	99.3	99.3	4	JF690890.seq
5	0.1	0.7	0.7	0.7		99.7	99.7	99.3	99.3	98.0	99.2	99.6	99.3	99.7	99.2	98.5	99.2	99.1	99.7	99.7	99.9	5	JN129482.seq
6	0.3	1.0	0.9	3.0	0.3		99.0	98.5	96.6	93.1	99.0	99.2	98.6	99.7	98.6	97.4	98.6	98.6	99.6	99.7	99.7	6	JX136950.seq
7	0.4	0.4	1.5	3.0	0.3	1.0		99.2	98.7	95.3	97.1	99.0	99.3	99.4	99.3	98.6	99.3	99.2	99.3	99.4	99.6	7	KF963260.seq
8	0.7	0.0	1.1	2.3	0.7	1.6	0.8		99.5	96.0	97.2	98.7	99.2	99.3	99.6	98.8	99.2	99.4	99.1	99.2	99.3	8	KT152814.seq
9	0.8	0.2	0.4	1.2	0.7	3.5	1.4	0.5		96.5	98.7	99.3	99.1	99.1	99.6	98.9	99.2	99.5	99.1	99.1	99.2	9	MH158272.seq
10	2.8	2.4	0.4	3.7	2.0	7.2	4.8	4.1	3.6		95.7	99.0	96.5	97.4	97.3	95.8	96.3	96.4	96.8	97.2	97.3	10	FJ997270.seq
11	0.8	0.3	0.7	2.2	0.8	1.0	3.0	2.9	1.4	4.4		98.9	99.1	99.1	99.4	98.2	99.1	98.7	98.8	99.2	99.2	11	GQ157251.seq
12	0.4	0.7	0.7	0.8	0.4	0.8	1.0	1.3	0.7	1.0	1.1		99.3	99.5	99.4	98.3	99.4	98.8	99.1	99.5	99.6	12	GX01.seq
13	0.6	0.7	0.8	1.2	0.7	1.4	0.7	0.8	0.9	3.6	0.9	0.7		99.4	98.9	98.3	100.0	98.8	99.3	99.4	99.4	13	GX02.seq
14	0.0	0.7	0.7	0.7	0.3	0.3	0.6	0.7	0.9	2.7	0.9	0.5	0.6		99.2	98.4	99.3	99.0	99.9	99.9	100.0	14	GX03.seq
15	0.8	0.1	0.3	0.2	0.8	1.4	0.7	0.4	0.4	2.7	0.7	0.6	1.1	0.8		99.2	98.9	99.8	99.1	99.1	99.2	15	GX04.seq
16	1.5	0.9	1.7	1.7	1.5	2.6	1.4	1.2	1.1	4.3	1.9	1.8	1.8	1.6	0.8		98.2	99.4	98.6	98.6	98.5	16	GX05.seq
17	0.7	0.8	0.9	1.2	0.8	1.4	0.7	0.8	0.8	3.8	0.9	0.6	0.0	0.7	1.1	1.8		98.8	99.3	99.3	99.3	17	GX06.seq
18	0.9	0.4	1.1	1.1	0.9	1.4	0.8	0.6	0.5	3.7	1.3	1.2	1.2	1.0	0.2	0.6	1.2		99.1	99.1	99.1	18	GX07.seq
19	0.0	0.8	1.0	1.1	0.3	0.4	0.7	0.9	0.9	3.3	1.2	0.9	0.7	0.1	0.9	1.4	0.7	0.9		100.0	100.0	19	GX08.seq
20	0.0	0.8	0.7	0.7	0.3	0.3	0.6	0.8	0.9	2.9	0.8	0.5	0.6	0.1	0.9	1.5	0.7	0.9	0.0		100.0	20	GX09.seq
21	0.0	0.7	0.6	0.7	0.1	0.3	0.4	0.7	0.8	2.8	0.8	0.4	0.6	0.0	0.8	1.5	0.7	0.9	0.0	0.0		21	GX10.seq
	1	2	3	4	5	6	7	8	9	10	11	12	13	14	15	16	17	18	19	20	21		

Figure S21. 16S rDNA Molecular Biological Identification Results of *Escherichia coli*. JF690890,JN129482,JX136950,KF963260,KT152814,MH158272,FJ997270,GQ157251 were *Escherichia coli* from different sources downloaded from NCBI as reference.
